# Genetic Diversity and Population Structure of Dülmen Wild, Liebenthal and Polish Konik Horses in Comparison with Przewalski, Sorraia, German Draught and Riding Horses

**DOI:** 10.3390/ani14152221

**Published:** 2024-07-31

**Authors:** Silke Duderstadt, Ottmar Distl

**Affiliations:** Institute of Animal Breeding and Genetics, University of Veterinary Medicine Hannover (Foundation), 30559 Hannover, Germany; silke.duderstadt@tiho-hannover.de

**Keywords:** genetic distance, heterozygosity, inbreeding, Merfelder Bruch, Dülmen, Croÿ, admixture, phylogeny, natural selection

## Abstract

**Simple Summary:**

Wild horses in the region around Dülmen in Westphalia, Germany, date back to the Middle Ages. These horses ranged the wooden and marsh area in small herds. The Dukes of Croÿ fenced these wild horses in 1856 in an area called Merfelder Bruch, near Dülmen, and managed this herd as a self-contained population. Stallions employed were from the Dülmen wild horse population and, in some breeding seasons, from other primitive horse populations. The Dülmen wild horse population has to cope with harsh natural conditions all year round. The Dülmen wild horse population consists of about 530 horses with around 380 mares. Each year, 2 to 3 stallions sire 50 to 60 foals. In the present study, we characterize, for the first time, the Dülmen wild horse population in comparison to warmblood, coldblood, and primitive horse populations using a highly informative microsatellite marker set. We found a high degree of genetic diversity in the Dülmen wild horse population. Genetic distance measures, principal component analysis, and Bayesian cluster analyses clearly demarcated the Dülmen wild horse population from the other 17 horse populations studied here. In addition, data supported the hypothesis of an early genetic divergence of Dülmen wild horses. The Dülmen wild horse population appeared as an invaluable resource to study the evolution of domesticated horses.

**Abstract:**

The objective of the present study was to analyze the genetic diversity, individual-based assessment of population structure, and admixture in the Dülmen wild horse population in comparison to warmblood, coldblood, and primitive horse populations. The Dülmen wild horse is kept as a unique horse population in the Merfelder Bruch near Dülmen in Westphalia, Germany, and since 1856 has been managed by the Dukes of Croÿ. The Dülmen wild horse population is exposed to the natural conditions of the Merfelder Bruch all year round without human interventions for feeding and veterinary care. In the present study, genetic diversity was estimated for 101 Dülmen wild horses using multilocus genotypic information from a set of 29 autosomal microsatellites and compared with 587 horses from 17 different horse populations. Dülmen wild horses maintained a high degree of genetic diversity, with an average observed heterozygosity of 0.68, a mean number of 6.17 alleles, and heterozygote deficit of −0.035. Pairwise genetic distances (F_ST_, Nei’s standard, and Cavalli-Sforza distances) were closest to German coldblood breeds, Polish Konik, and Icelandic horses and most divergent from Sorraia and Przewalski’s horses. Neighbor joining dendrogram and PCA plots showed a clear distinction of Dülmen wild horses from other populations, particularly from Przewalski horses. Posterior Bayesian analysis confirmed clear differentiation from other horse populations without an admixture pattern and a high membership index (0.92). It was possible to distinguish Dülmen wild horses from Dülmen and Polish Konik horses. In conclusion, Dülmen wild horses show a notable separation from other German horse breeds and primitive horse populations and may serve as a resource to study evolution of equine domestication.

## 1. Introduction

The earliest reports on the Dülmen Wild Horses in the region around Dülmen in Westphalia, Germany, date back to 1316 [1]. In the Middle Ages, these horses ranged the wooden and marsh area in small herds. Since 1856, these wild horses were fenced in the Merfelder Bruch near Dülmen in Germany by the Dukes of Croÿ and managed as an independent population. Stallions employed were from the Dülmen wild horse population and, in some breeding seasons, stallions from other breeds, such as Mongolian, Exmoor, and Polish Konik. However, no fillies or broodmares from other horse breeds were introgressed into the Dülmen wild horse population since its foundation, as early as 1856 [1]. The primary objective is to maintain the Dülmen wild horse population under harsh natural conditions in order to conserve the genetic ability to cope with natural selection pressure. The present-day population involves approximately 530 horses, with around 380 mares of reproductive age and 50–60 foals each year, as well as 80–100 female horses aged 1–3 years (Supplementary Materials Figures S1 and S2). Each year, the one-year-old colts are removed from the herd and sold by public sale on the last weekend in May. Two to three stallions are employed for natural service each breeding season for 2–3 months. The number of pregnant mares is regulated through the time span the stallions are allowed to sire mares. After each breeding season, the stallions are kept in a separate group from the mare herd. Approximately 3–6 stallions are kept on their own pasture, which is a few kilometers away from the area with the mares, foals, and young horses in the Merfelder Bruch. To prevent mating of stallions with their daughters, stallions are employed for a maximum of three consecutive breeding seasons. After this time, stallions leave the Merfelder Bruch and do not return either to the mare herd or to the stallion pasture. The Dülmen wild horse herd is unique as no females leave the Merfelder Bruch for breeding outside this herd. Dülmen wild horse stallions are employed to sire mares from other domesticated pony breeds. These progeny are registered as Dülmen horses in a herdbook separate from the Dülmen wild horse (Supplementary Materials Figure S3).

The Liebenthal horses originate from a breeding program initiated by Jürgen Zutz in Bavaria in 1960 [2]. The aim was to breed a horse with a similar appearance to the extinct tarpan (*Equus ferus ferus*). Polish Konik, Dülmen, Przewalski, and Norwegian horses were used for establishing the breeding stock. A herd of Liebenthal horses is maintained in the Schorfheide, near Liebenthal in Brandenburg, Germany (Supplementary Materials Figure S4). Stallions are assigned to specific groups of mares throughout the year. Colts and young stallions are separated from the herd. Liebenthal horses are small and robust horses with dun color and primitive markings [3].

The Polish Konik horses are Polish native horses, which have their origin in eastern Poland and may have genetic relationships to the extinct tarpan (*Equus ferus ferus*), which lived on the steppes of southeast Europe and Asia [4,5]. The breeding history of the Polish Konik horses started as early as 1923 [5,6,7,8,9,10]. In 1936, the first reserve was established, and, in 1954, a stud farm in Popielno was founded to collect all Polish Konik horses in one place and to manage breed conservation based on a studbook [5,6,7,8,9,10,11,12,13,14,15,16]. The first studbook for Polish Konik horses was released in 1962 [6,11]. The breeding program within a closed studbook [13] has the objectives to maintain all six paternal [14,16] and as many as possible of the 35 maternal lineages known in 1962 [12,15,16]. However, only 16 maternal lines have survived in the present-day population [15,16]. Polish Konik horses are perfectly adapted to harsh natural conditions and suitable for maintenance of valuable landscapes [5,8,10,11,17]. In the 2022 Polish Konik studbook, there are approximately 1760 mares, 183 stallions, and 1540 foals registered [16].

The objective of the present study is to characterize the genetic diversity of the Dülmen wild horse in comparison to modern horse breeds and other primitive breeds including the Dülmen, Liebenthal, Sorraia, Polish Konik, and Przewalski horse. We used 30 autosomal microsatellite markers (STRs) and included data from a previous study on the genetic diversity of 12 horse populations [18]. In the present study, the genotype data of the Dülmen wild, Dülmen, Liebenthal, and Polish Konik horses, as well as two coldblood breeds, were supplemented. The marker set is highly informative for primitive horse breeds, well suited for analyses of genetic diversity, and population structure and phylogeny. In addition, it allows comparisons with other populations already genotyped with this set of STRs [18] as well as with previous studies using STRs [6,14,15,19,20]. The samples of the other primitive and cold-blooded horse breeds from our previous study [18] used for comparison were no longer available. It is assumed that the German draught horse breeds originated from local working breeds of the 16th to 18th centuries and were not intermixed with oriental or Arabian horses [18]. Arabian horses had a major influence on all European riding horses, and Hanoverians are the largest population of riding horses in Germany [18]. Due to its large genetic distances to all other populations, the Przewalski horse was included as an outgroup [18]. Therefore, we should be able to investigate whether the Dülmen wild horse is possibly related to German draught horse breeds through earlier local populations or was influenced by oriental horses, or whether it has more distant common ancestors with Exmoor ponies and/or Icelandic or Sorraia horses.

## 2. Materials and Methods

### 2.1. Ethical Approval

The study was approved by the Institutional Review Board of the University of Veterinary Medicine Hannover (Foundation) and the German Federal State offices from North Rhine-Westphalia (registration number 8.84-02.05.20.12.066) on 17 April 2012, Rhineland-Palatinate (registration number 23 177-07/A 15 20-002 OEW) on 21 August 2015, Hesse (registration number V54-19c20/15-V/Anz. 1014) on 4 November 2015, Lower Saxony (registration number 33.9-42502-05-15A574) on 21 September 2015, and Brandenburg (registration number 2347-A-19-1-2014) on 15 December 2014.

### 2.2. Sample Collection

Hair root samples were collected from Dülmen wild horses (n = 101), Dülmen horses (n = 27), Liebenthal horses (n = 47), Polish Konik horses (n = 26), Altmaerkisch coldblood (n = 32), and Friesian horses (n = 47). The samples of the Dülmen Wild Horse were taken from three different birth year cohorts (2011, 2012, and 2013) in the Merfelder Bruch. In the preceding covering periods, six different stallions were employed. Per season, two (2011) or three (2010 and 2012) stallions were breeding approximately 60–80 mares. Only one stallion was used in all three consecutive seasons. All stallions were selected from the herd in the Merfelder Bruch. The distribution of the sampled horses by their sex and origin is given in Supplementary Materials Table S1. In addition, genotypic data from Hanoverian warmblood (n = 47), Arabian (n = 26), Sorraia (n = 23), Icelandic (n = 45), Exmoor (n = 20), Przewalski (n = 21), and German draught horses, including South German (n = 45), Rhenish German (n = 46), Black Forest (n = 45), Schleswig (n = 45), Mecklenburg (n = 22), and Saxon Thuringa draught (n = 23) [18], were added to the present analysis. In total, the dataset comprised 688 horses of 18 different horse populations.

### 2.3. DNA Extraction and Microsatellite Analysis

All laboratory work was carried out following sample collection. Genomic DNA was extracted from hair root samples using proteinase K digestion and ethanol extraction. We chose microsatellite markers with a high heterozygosity level, high number of different alleles, and good technical properties for amplification from 30 different autosomes according to Aberle et al. [18]. This marker set includes the markers AHT34, ASB17, COR007, COR017, COR018, COR022, COR024, COR045, COR056, COR058, COR069, COR070, COR071, COR082, HMS03, HMS07, HTG03, HTG06, LEX07, LEX33, LEX34, LEX63, LEX68, LEX73, SGCV16, SGCV28, TKY19, CA425 (UCDEQ425), UM011, VHL20, and VHL209. Due to the low heterozygosity and the technical properties during amplification, the only marker from Aberle et al. 2004 [18] not used in the present study was HMS003. All 30 other markers are identical to the marker set of Aberle et al. 2004 [18]. We developed this marker set as it was more informative and covered each autosome [18]. Furthermore, for phylogenetic and Bayesian analyses, more than 20 markers should be applied [18]. Multiplex PCRs with fluorescently labelled primers were used for amplification. Each 10 µL PCR reaction tube contained 20 ng genomic DNA, 1.4 µL 10× incubation PCR buffer with MgCl_2_ (MP Biomedicals, Illkirch, France), 0.5 µL DMSO, 100 µM each dNTP, 0.5 U *Taq*-DNA polymerase (MP Biomedicals), 5′ IRD700 or IRD800 (IRD: infrared dye) labelled forward primer, and unlabeled reverse primer. The amplification was carried out in PTC-100™ thermocyclers (MJ Research, Watertown, MA, USA) under the following conditions: an initial denaturation step at 94 °C for 4 min followed by 36 cycles at 94 °C for 30 s, maximum annealing temperatures for 60 s (Supplementary Materials Table S2), and a final extension of 30 s at 72 °C. The dilution of PCR products with formamide loading dye in ratios from 1:4 to 1:16 was determined empirically and carried out prior to size fractionating on 6% denaturing polyacrylamide (Rotiphorese Gel 40, Carl Roth, Karlsruhe, Germany) sequencing gels. Gel electrophoresis was performed using a LI-COR 4300 DNA analyzer (LI-COR Biosciences, Lincoln, NE, USA) connected to computers on a network. Gel images showing the amplified fragments of the STR markers, one per lane, were stored on the computer. Reference markers with known fragment lengths and 100 bp molecular weight standards (LI-COR Biosciences) on each gel were used to calibrate allele sizes. The printouts of the gel images were analyzed manually. SAGA GT^TM^ software (LI-COR Biosciences) was used to confirm the amplified product sizes of the markers. The allele sizes of the STR markers were manually transferred to an Excel spreadsheet together with the marker name, gel run, and animal identification. 

### 2.4. Statistical Analysis

#### 2.4.1. Genetic Diversity, Population Differentiation and Phylogeny

Identification of possible genotyping errors due to null alleles was performed using Micro-Checker software [21]. Deviations from Hardy–Weinberg Equilibrium (HWE) were determined across all loci in each population using Chi-square tests (SAS/Genetics, version 9.4, Statistical Analysis System Institute, Cary, NC, USA, 2020). In addition, we determined polymorphism information content (PIC) and allelic diversity (AD) for each marker over all populations. Allele frequencies, the number of alleles (N_A_), observed (H_o_) and expected (H_e_) heterozygosity, number of private alleles (PA), and Wright’s F_IS_ [22] were calculated using GENEALEX 6.5 [23]. Molecular genetic relationships among populations were derived using Wright’s F_ST_ [22], Nei’s standard genetic distance (D_S_), and CavalliSforza chord distance (D_C_). Those three measures of population differentiation are supposed to provide different information. F_ST_ is not based on an evolutionary model, and it has been shown to be appropriate for the analysis of closely related populations where drift is the main force of differentiation. The chord distance (D_C_) by Cavalli-Sforza [24] is one of the best qualified for use with populations of intermediate divergence time as represented by breeds worldwide and in the breeds under study. However, standard genetic distance (D_S_) of Nei [25] is the more frequently used distance, and this was calculated to have the possibility of comparing our results with those of other studies. The population differentiation measures D_C_, D_S_, and F_ST_ were calculated using Microsatellite analyzer (MSA) 4.05 [26]. The matrices with the population differentiation measures F_ST_, D_C_, and D_S_ were employed for principal component analyses (PCA) using SAS, version 9.4 (Statistical Analysis System Institute, Cary, NC, USA, 2020) and displayed as scatter plots with SAS, version 9.4. The neighbor-joining tree topology was obtained with the PHYLIP software version 3.65 [27] using Nei’s distance. Bootstrap values were computed over 1000 replicates, and a consensus tree was drawn using TREEVIEW 1.6 [28]. Molecular kinship distance (D_k_) accounts for the allele frequencies in the founder populations and is considered as an evolutionary mid-term measurement [29]. R_ST_ is based on the mutation rate of marker length under the assumption of a strict stepwise mutation model (SMM) and accounts for the long-term evolution of populations.

#### 2.4.2. Assessment of Population Structure and Admixture

The genetic structure of the populations and degree of admixture of the 18 populations was inferred with posterior Bayesian clustering methods developed by Pritchard et al. [30] and Falush et al. [31,32,33] in the software STRUCTURE 2.3.4. STRUCTURE uses a Markov chain Monte Carlo (MCMC) approach to estimate the most likely membership of an individual to a preassigned number of clusters (K). This likelihood is calculated using the natural logarithm of the probability (P) of the observed genotypic array (G), given the preassigned number of clusters in the data set ln P(G|K). The most likely number of clusters corresponds to the maximum of the likelihood function ln P(G|K). We ran STRUCTURE in 10 independent repetitions for each K from 1 to 20 to obtain a representative set of values of the likelihood function ln P(G|K). The number of iterations in each run was 10^6^ after a burn-in length of 10^5^ iterations. We chose an admixture model with a correlated allele frequencies model and a uniform prior. Individual admixture alpha was set to be the same for all clusters. We employed the software STRUCTURESELECTOR [34] to calculate the mean ln P(K) and its standard deviation, ln′(K), |ln″(K)|, and ΔK statistics [35] from 10 repeated runs. In addition, the same output datasets from STRUCTURE were used to derive the four alternative statistics (MedMed K, MedMean K, MaxMed K, and MaxMean K), which were proposed by Puechmaille (2016) [36]. These alternative statistics use either the median (Med) or the maximum (Max) of the proportion of membership for each cluster (K = 1–20), calculated from the 10 replicates. The threshold value for the membership coefficient above which a population was considered as a member of a cluster was set at 0.7. The maximum of these values for all 20 pre-assigned clusters was chosen as an estimate of the number of clusters that can be distinguished in the present dataset. Graphical representation of the results, including the population cluster stratifications with CLUMPAK [37], mean ln P(K), standard deviation (sd) of ln P(K), ln′(K), |ln″(K)|, ΔK, MedMed K, MedMean K, MaxMed K, and MaxMean K were displayed using STRUCTURESELECTOR [34].

## 3. Results

### 3.1. Characteristics and Genetic Diversity of the Microsatellite Markers

LEX073 showed a significant deviation from HWE in more than one population and was excluded from all further analyses (Supplementary Materials Table S3). After Bonferroni correction, no significant deviation from HWE was given at any further marker. There were no loci with null alleles. The least polymorphic marker was COR022 with 4 alleles (N_A_) per locus over all samples and the most polymorphic marker was ASB017 with 19 alleles per locus (Supplementary Materials Table S4). The mean N_A_ was 10 alleles. The sum of all alleles over all loci amounted to 300. Observed heterozygosity (H_o_) ranged from 0.360 (HTG006) to 0.786 (COR058). Measures of population differentiation Wright’s F_ST_ and G_ST_ ranged from 0.143 (COR045) to 0.249 (COR056) and from 0.130 (COR045) to 0.237 (COR056), respectively. Measures of marker informativeness (PIC) and allelic diversity (AD) ranged from 0.416 (HTG006) to 0.901 (COR058) and from 0.435 (HTG006) to 0.908 (COR058), respectively. Average PIC and AD across all markers were 0.744 and 0.771, respectively.

### 3.2. Genetic Diversity, Genetic Distances and Phylogeny

Observed heterozygosity in Dülmen wild horses (0.681) was greater than in Sorraia (0.523) and Przewalski’s horses (0.488), but comparable to German coldblood horses (0.633–0.703) (Table 1). Allele richness was 6.17 and expected heterozygosity was 0.658 in Dülmen wild horses. Only Hanoverian warmblood, Icelandic horses, and South German Coldblood showed a higher mean number of alleles (6.69, 6.45, and 6.31, respectively). Two private alleles were found in Dülmen wild horses. F_IS_ coefficients varied around zero and a slightly negative value was found for Dülmen wild horses (−0.035).

Pairwise F_ST_ estimates among the 18 horse populations are shown in Supplementary Materials Table S5. The largest differentiation was seen among Dülmen wild horses and Sorraia (0.158), as well as Przewalski’s horse (0.167). Genetic differentiation with F_ST_ estimates was moderate between Dülmen wild horses and German coldblood breeds (0.062–0.081), Polish Konik (0.076), as well as Icelandic horses (0.067). The smallest differentiation was to Dülmen horses (0.048), which are descendants from stallions of Dülmen wild horses.

Nei’s standard genetic distances (Ds) (Supplementary Materials Table S6) and Cavalli-Sforza chord distances (D_C_) (Supplementary Materials Table S7) gave similar results compared to F_ST_ estimates. For Dülmen wild horses, the lowest estimates for Ds and D_C_ were found when compared with Dülmen horses (0.110 and 0.287, respectively) and highest to Sorraia (0.425 and 0.571) and the Przewalski’s horse (0.491 and 0.612, respectively). 

**Figure 1:**
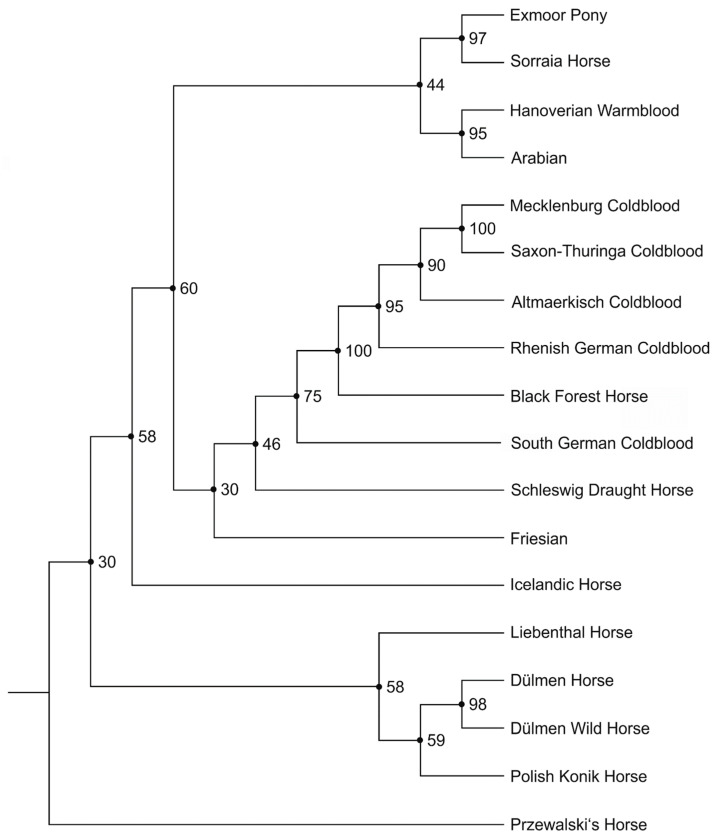
Nei’s distance (D_s_) neighbor-joining dendrogram including 18 horse populations with the Przewaslski’s horse as an out-group. The tree was estimated from 1000 bootstraps. Bootstrap values are in percentages.

The neighbor-joining dendrogram of the Nei’s distance Ds resulted in four groups, assuming the Przewalski’s horse as an out-group (Figure 1). 

Dülmen wild horses formed an own branch with Polish Konik, Liebenthal, and Dülmen horses, which separated from all other remaining populations very early in the dendrogram. Next, the Icelandic horses split off and formed their own branch. The next branch contained all German coldblood breeds with Friesian horses, and the last branch contained a group with each two subtrees including Hanoverian and Arabian in one subbranch and Exmoor and Sorraia in the other one. The clusters had a support from 30 to 100%.

### 3.3. Principal Components Analysis

**Figure 2:**
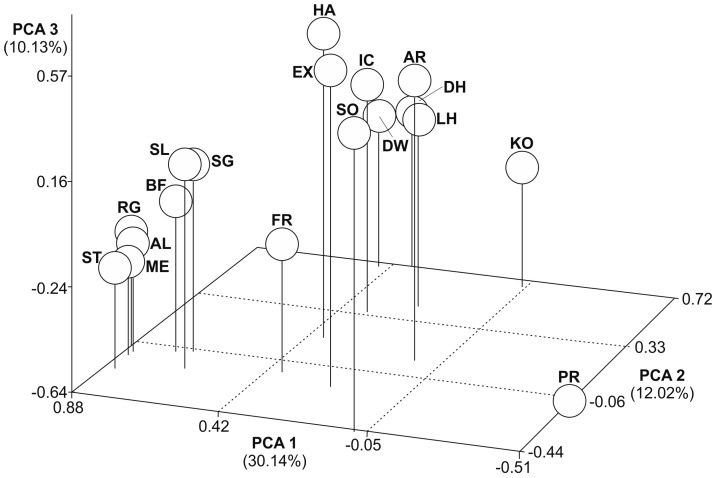
Principal component analysis of 18 populations based on D_C_. Each population was plotted into scatter plot. PCA 1 accounts for 30.14% of the variation, PCA 2 for 12.02%, and PCA 3 for 10.13%. Abbreviations in Table 1.

**Figure 3:**
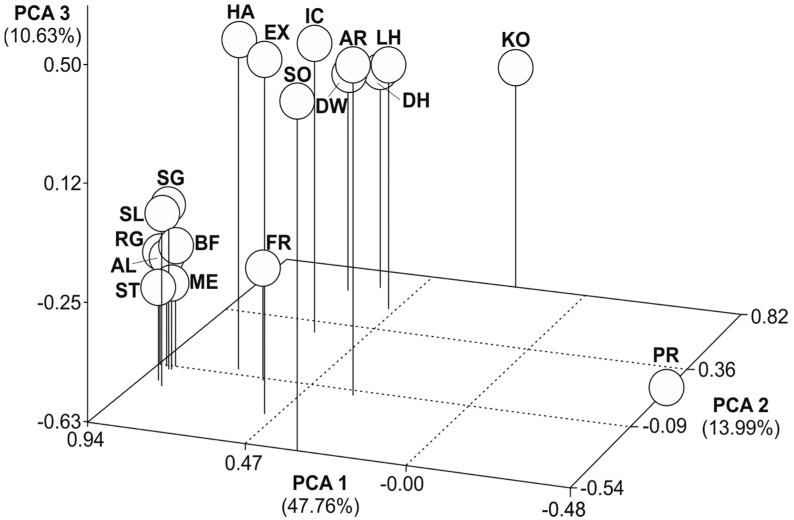
Principal component analysis of 18 populations based on D_S_. Each population was plotted into scatter plot. PCA 1 accounts for 47.76% of the variation, PCA 2 for 13.99%, and PCA 3 for 10.63%. Abbreviations in Table 1.

**Figure 4:**
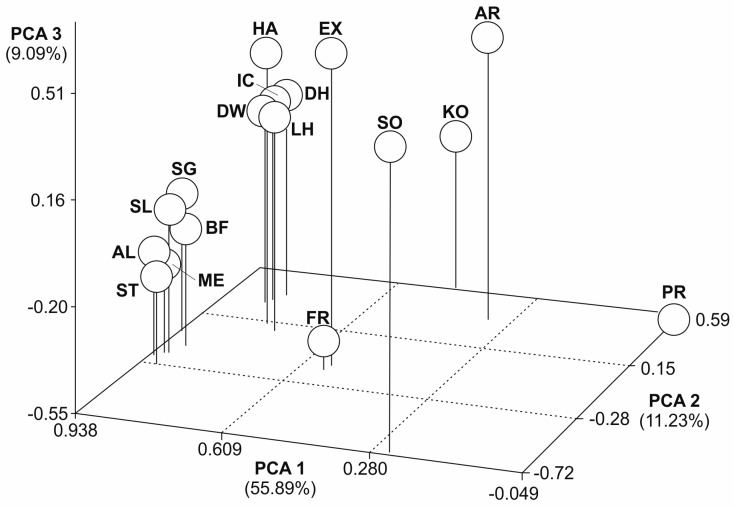
Principal component analysis of 18 populations based on F_ST_. Each population was plotted into scatter plot. PCA 1 accounts for 55.89% of the variation, PCA 2 for 11.23%, and PCA 3 for 9.09%. Abbreviations in Table 1.

Three-dimensional scatter plots of the principal component analysis (PCA) showed the molecular genetic relationships among the 18 horse populations based on D_C_, D_S_, and F_ST_ distance estimates (Figure 2, Figure 3 and Figure 4). Variance explained through the first three PCAs ranged from 30 to 10% (D_C_), 48 to 11% (D_S_), and 56 to 9% (F_ST_). All plots showed a similar pattern, with three main groups. German draught horse breeds form a distinctive group in all three plots. The Przewalski’s horse population was placed far away from all other horse populations. Friesian, Sorraia, and Polish Konik were also outside the other populations. All further populations were scattered in-between the German coldblood breeds and the Przewalski’s horse population without forming subclusters with close relationships. The Dülmen wild horse population was differentiated mainly through the second and third principal component. 

### 3.4. Population Structure and Admixture

**Figure 5:**
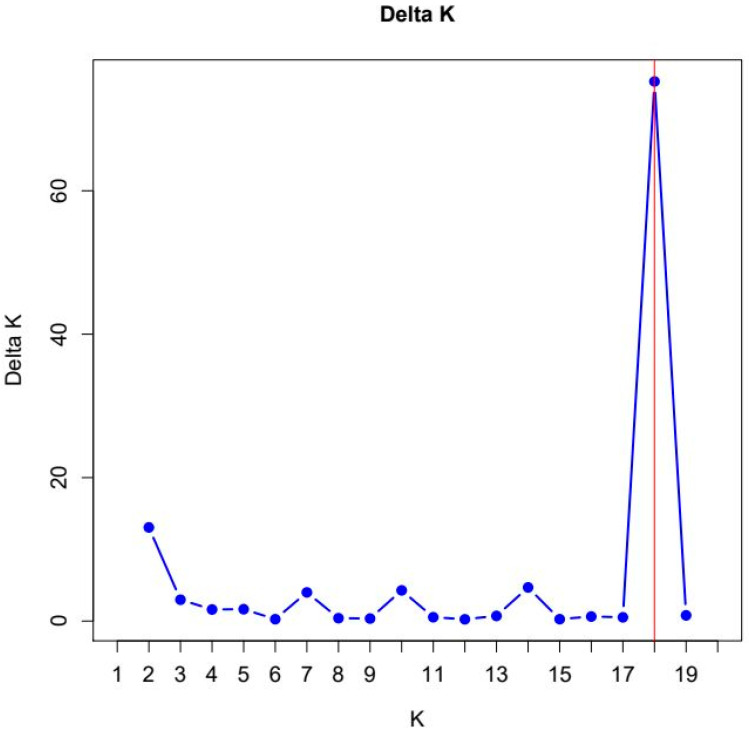
Plot of ΔK (Delta K) values from 10 independent STRUCTURE runs for K = 2–19. The maximum value is indicated by a red vertical line.

The most likely number of clusters is obtained where ΔK reaches its maximum between K = 2 and 19 (Figure 5 and Supplementary Materials Table S8). With K = 18, ΔK reaches its maximum value with 75.24. The mean of the likelihood function also reaches its maximum at K = 18 (Supplementary Materials Figure S5). For runs with K smaller or larger than 18, the value for ΔK reached much smaller values. 

**Figure 6:**
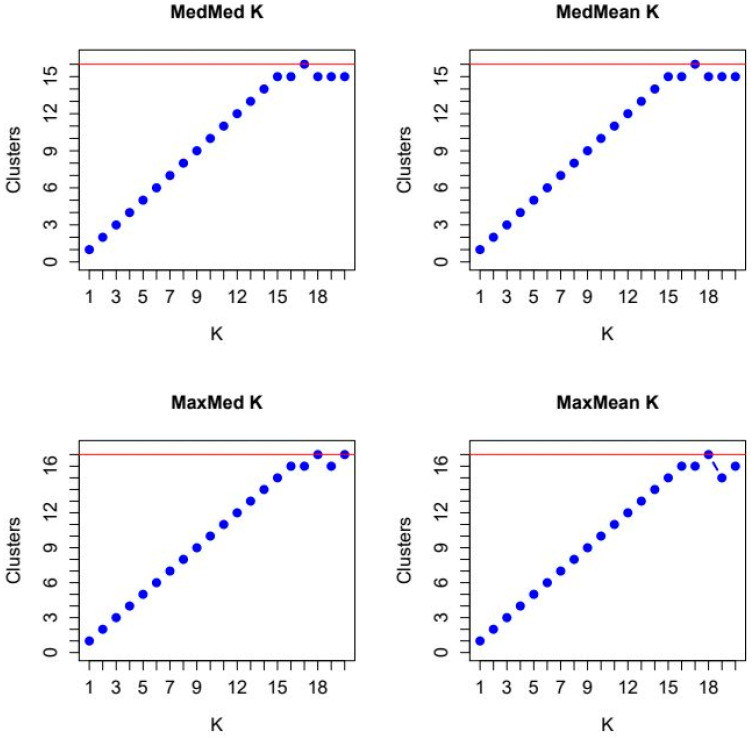
Plots of the medians (Med) and means (Mean) of the median (Med) and maximum (Max) number of inferred proportions of memberships to clusters with a threshold of 0.7. The red line indicates the maximum values reached by the MEDMEDK, MEDMEAK, MAXMEDK and MAXMEAK statistics.

For the statistics MEDMEDK and MEDMEAK, we obtained maxima for K = 17 with 16 clusters, and for the statistics MAXMEDK and MAXMEAK, we obtained maxima for K = 18 with 17 clusters (Figure 6). The reason for these results was the distribution of the membership coefficients for the assumed clusters. For K = 17, one cluster had only membership coefficients <0.7 for the 9/10 independent STRUCTURE runs (Supplementary Materials Table S9). For K = 18, in 1/10 STRUCTURE runs, Polish Koniks were split into two clusters with membership coefficients of 0.87 and 0.1, respectively, and these clusters were not shared by other breeds with membership coefficients >0.7 (Supplementary Materials Table S10). Therefore, the number of clusters with membership coefficients above the threshold of 0.7 for MEDMEDK and MEDMEAK across all 10 independent runs was 16 and for MAXMEDK and MAXMEAK 17. For K = 17–18, the coldblood breeds Altmaerkisch, Mecklenburg, and Saxon-Thuringan shared the same cluster in all 10 independent STRUCTURE runs, except for one STRUCTURE run with K = 18 (Supplementary Materials Tables S9 and S10). For K = 17, the membership coefficients for Altmaerkisch were on average 0.9 and for Mecklenburg and Saxon-Thuringan on average 0.8. Dülmen wild horses had membership coefficients at 0.92 for K = 17–18. The highest membership coefficients were shown by Friesian horses, with >0.95, and Sorraia, with > 0.96, across all 10 independent runs for K = 17–18.

**Figure 7:**
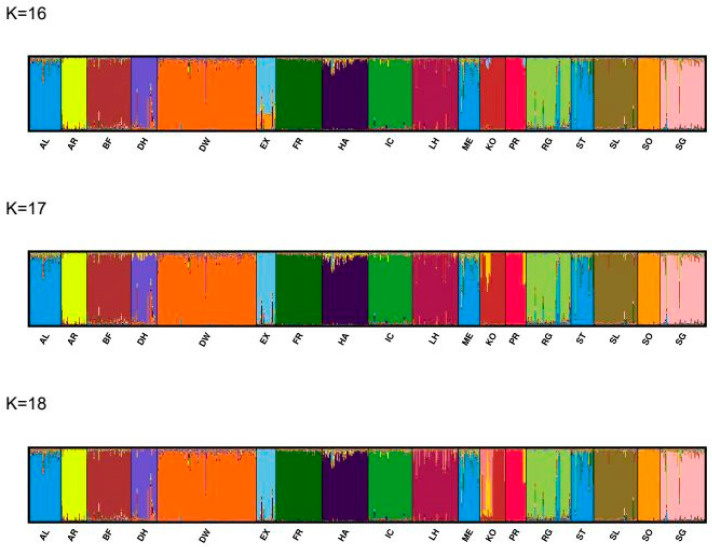
The figure shows the major modes of CLUMPAK plots of K = 16–18 from 10 independent STRUCTURE runs from K = 1–20 using 688 horses from 18 breeds. Each individual is represented by a vertical line divided into K colors, where K is the number of clusters assumed and the colors show the consensus solutions for individual proportions of cluster memberships. Populations are separated by black lines. Abbreviations in Table 1.

We displayed the major modes from the post-processed population structure results for K = 16–18 in Figure 7 and for K = 2–18 in Supplementary Materials Figure S6 based on CLUMPAK [34,37]. 

For K = 2, one cluster included the Dülmen horse, Dülmen wild horse, Liebenthal, and Polish Konik, and the second cluster contained all other horse populations (Supplementary Materials Figure S6). For K = 4, the Dülmen horse, Dülmen wild horse, and Polish Konik horse were grouped in the same cluster and this cluster remained up to K = 7. With K = 8, Polish Konik horses started to separate from Dülmen horses and Dülmen wild horses, showing variable admixtures with other populations. From K = 13, Polish Konik formed their own separate cluster. Dülmen horses and Dülmen wild horses are divided into two separate clusters from K = 16. From K = 12–15, Dülmen horses show increasing admixture with other populations. With K = 16, all Dülmen wild horses were assigned to one cluster and were clearly separated from Dülmen horses. The Altmaerkisch, Friesian, Mecklenburg, Rhenisch German, Saxon-Thuringan, Schleswig, and South German coldblood were assigned to one cluster from K = 3. Coldblood breeds started to split into more than one cluster from K = 7. Admixture patterns were particularly seen in the German coldblood breeds Altmaerkisch, Mecklenburg, Rhenisch German, Saxon-Thuringan, and South German as well as in Exmoor, Liebenthal, and Polish Konik horses. Przewalski’s horse appeared as an independent group from K = 7. The CLUMPAK plots indicated that Altmaerkisch, Mecklenburg, and Saxon-Thuringan in particular, and, to a lesser extent, also Rhenisch German coldblood horses, were admixtures between these coldblood breeds.

## 4. Discussion

The molecular genetic data shown represent the genetic characterization of the Dülmen wild horse, which has never been described before. We identified their genetic relationships between other primitive and modern horse populations. The Dülmen wild horse demonstrated high values for N_A_ and H_o_ and low values for F_IS_. The mean H_o_, H_e_, N_A_, and F_IS_ reported in the literature for other horse breeds [6,7,14,15,19,20,38,39,40,41,42] mostly varied in the same range as our data, but the genetic diversity retained by the Dülmen wild horses was in the upper range compared to other primitive horse populations. Mongolian horse populations reached average heterozygosities of 0.75–0.77, which were higher than in Hanoverian horses, whereas Japanese populations had lower values, ranging from 0.34 to 0.66 [41]. The population of the Polish Konik horses in our study showed a smaller He and N_A_ than in the study of Mackowski et al. [6], who employed a larger sample size of Polish Konik horses and a smaller set of microsatellites. The degree of genetic diversity of the Polish Konik horses studied is lower than that of the Dülmen wild horse. 

The genetic differentiation between the horse populations in the present data was studied using different approaches, such as genetic distances, principal component analysis, and Bayesian clustering. All three genetic distance measures based on genetic relationships, including Wright’s F_ST_, Nei’s standard genetic distance (D_S_), and Cavalli-Sforza chord distance (D_C_), produced similar results and differentiated the Dülmen wild horse population from all other populations. In line with the genetic distance measures, PCAs, with their graphic representation and neighbor-joining dendrograms based on Nei’s distance, also clearly demarcated the Dülmen wild horse population from the 17 other horse populations studied. There was evidence for especially large genetic distances to Przewalski and Sorraia horses. Moderate genetic distances were found for Arabian, Hanoverian, Exmoor, and Friesian, and even smaller genetic distances to Icelandic, Liebenthal, Polish Konik, and German coldblooded horses. The neighbor-joining dendrogram with Nei’s distance and the Przewalski’s horses as outgroup supported the topology with a private cluster for Dülmen wild, Dülmen, Liebenthal, and Polish Konik horses. This cluster branched off before a common node for the branches of the other 13 horse populations was estimated. The closer genetic relationships among Dülmen wild and Dülmen horses stems from the use of Dülmen wild horse stallions to sire progeny from other domesticated pony breed mares. A gene flow from modern pony breeds via the maternal path into the Dülmen wild horse population is not possible due to the strict breeding regulations in place since 1840. Between 1957 and 2004, nine different Polish Konik stallions were used in the Merfelder Bruch herd for covering and 6/9 Polish Konik stallions had sons who were also employed as stallions in this herd. The most influential sires were progeny from Kurs, born on 4 December 1960, breeding license number 5196, and Tulipan, born 21 March 1979, breeding license number 9 G Bł, from the Gorja sire line (PZHK Pedigree database, https://baza.pzhk.pl/en/horse/id/4914.html, accessed on 17 May 2024). In this way, a gene flow from Polish Konik into the Dülmen wild horse population was made possible. In the present study of Dülmen wild horses, only two foals were sired by an F_1_-crossbred stallion of the Gorja sire line with a Dülmen wild horse mare. All other sires of the Dülmen wild horses in our study were bred in the original Dülmen wild horse herd in the Merfelder Bruch. 

Icelandic horses diverged from the common node for the 13 horse populations and left a common node for German coldblood, Friesian, Arabian, Hanoverian, Exmoor, and Sorraia. Cluster topology indicated a common development of German coldblood breeds and Friesian horses, whereas Sorraia and Exmoor may share some genetic similarity. 

Posterior Bayesian estimation [30,31,32,33] and postprocessing results from CLUMPAK [37] supported an early genetic divergence among Dülmen wild horses together with Polish Konik, Liebenthal, and Dülmen horses from all other horse populations under study. The influence of the gene flow from Dülmen wild horses into Dülmen and Liebenthal horses caused the early divergence of Dülmen and Liebenthal horses. This may be expected as Dülmen horses are mainly F_1_-progeny from other domesticated pony breed mares and Dülmen wild horse stallions. This joint divergence between Dülmen wild horses and Dülmen horses supports the hypothesis of an early divergence of Dülmen wild horses and of a self-standing population without an intermixing with domestic breeds in former times. A similar hypothesis may be claimed for the Polish Konik horse as Polish Konik share the same cluster with the Dülmener populations up to K = 7.

The breed history of Dülmen wild horses seems less complex compared to the modern breeds, where intermixing among local populations and upgrading with stallions of Thoroughbreds, Arabian, or Spanish breeds was a common feature [18,20]. Due to their uniqueness, allelic richness expressed through observed and expected heterozygosity, and mean number of alleles, conservation of the Dülmen wild horse population under natural harsh conditions should be maintained. Introgression of genes from modern horse breeds through stallions must be strictly avoided. F_1_-Dülmen wild horse by Polish Konik (B_1_) stallions backcrossed for more than six generations should ensure a small increase of the inbreeding rate and maintain the genetic heritage of the Dülmen wild horse. Further purebred Polish Konik stallions should only be used in very limited numbers and breeding seasons to produce backcross stallions. Another option to maintain genetic diversity as large as possible in the Dülmen wild horse population, without continued intermixing with Polish Konik horses, should be to preselect potential stallions according to their genetic relatedness with each other and to the breeding herd estimated via the male progeny, as well as according to their own genetic diversity in molecular genetic markers.

## 5. Conclusions

In conclusion, we characterized a unique horse population with a very distinguished history for more than 180 years in Dülmen, Westphalia, Germany, using a very informative set of microsatellites. A high genetic diversity was maintained in the Dülmen wild horse population. This herd is in the private hands of the Dukes of Croÿ. The Dülmen wild horse population is an invaluable resource for studying evolution and disease dispositions [43] of domesticated horses in Central Europe. This is the first report on this horse population. Previous studies included domesticated Dülmen horses or ponies [6,16,44], and thus the uniqueness and the potential value of the wild herd for phylogenetic studies was obscured.

The results of this study demonstrated the high genetic diversity retained in the Dülmen wild horse and supported an early genetic divergence of the Dülmen wild and Polish Konik horse from modern, coldblooded, Icelandic horses, and other primitive horse populations. Genetic distance measures, neighbor-joining dendrograms based on Nei’s distance, Bayesian clustering, and postprocessing for ΔK and consensus membership coefficients across 10 independent runs and 20 clusters showed that Dülmen wild horses can be differentiated from other horse populations. A limitation of this study may be seen in that fillies and mares cannot be accessed for sampling as this horse population is not tame and capturing of mares for sampling is not possible due to the risk of injury of humans and horses and the expected adverse effects on the natural behavior of this herd. Conclusions on the genetic diversity of this horse population can therefore only be drawn from the male generation. There are no records of mating, and the foals are not registered, as this herd is kept without any disturbance or human intervention to preserve the natural behavior of the Dülmen wild horses. Continued monitoring of the development of genetic diversity using STR markers and high-density DNA arrays can be recommended. Planned breeding stallions should be examined for their genetic relationship to the stallions used and an inbred load matrix [45] should be derived from the genotype data of the male progeny. The time span for these analyses should cover the last 10–20 birth year cohorts, as mares reproduce into old age.

## Figures and Tables

**Table 1:** Sample size (N), observed (H_o_) and expected (H_E_) heterozygosity, mean number of alleles per locus (MNA), total number of private alleles (PA), and the heterozygote deficit (F_IS_) averaged over 29 microsatellites in 688 horses from 18 horse populations. Draught horse populations include AL, BF, ME, RG, ST, SL, and SG.

Population		N	H_o_	H_e_	MNA	PA	F_IS_
Altmaerkisch Coldblood	AL	32	0.633	0.642	5.38	1	0.016
Arabian	AR	26	0.576	0.562	4.34	2	−0.016
Black Forest Horse	BF	45	0.691	0.653	5.86	-	−0.056
Dülmen Horse	DH	27	0.720	0.660	5.55	1	−0.094
Dülmen Wild Horse	DW	101	0.681	0.658	6.17	2	−0.035
Exmoor Pony	EX	20	0.614	0.567	4.41	2	−0.071
Friesian	FR	47	0.542	0.538	4.31	2	−0.003
Hanoverian Warmblood	HA	47	0.738	0.734	6.69	7	−0.008
Icelandic Horse	IC	45	0.719	0.729	6.45	3	0.015
Liebenthal Horse	LH	47	0.673	0.665	5.14	2	−0.015
Mecklenburg Coldblood	ME	22	0.636	0.640	5.38	-	0.016
Polish Konik Horse	KO	26	0.639	0.569	4.10	1	−0.125
Przewalski Horse	PR	21	0.488	0.562	4.24	9	0.131
Rhenish German Coldblood	RG	46	0.702	0.685	6.07	-	−0.025
Saxon-Thuringa Coldblood	ST	23	0.686	0.647	5.17	2	−0.060
Schleswig Draught Horse	SL	45	0.693	0.679	5.45	1	−0.023
Sorraia Horse	SO	23	0.523	0.516	3.41	-	−0.018
South German Coldblood	SG	45	0.703	0.701	6.31	2	−0.006

## Data Availability

Restrictions apply to the availability of these data. Samples were obtained from Dülmen wild horses in the Merfelder Bruch with allowance of Rudolph Herzog von Croÿ, from Liebenthal horses in the Wildpferdegehege, Liebenwalde, from Dülmen horses in the Stiftung Naturschutzpark Lüneburger Heide, Hof Tütsberg, horse owners and breeders and are available from the authors at a reasonable request and with the permission of the horse owners.

## Supplementary Materials

The following supporting information can be downloaded at: https://www.mdpi.com/article/10.3390/ani14152221/s1, Figure S1. Photograph of a Dülmen wild horse mare in the Merfelder Bruch. Figure S2. Photograph of Dülmen wild horse stallions born in the herd of the Merfelder Bruch. Figure S3. Photograph of a Dülmen horse gelding. Figure S4. Photograph of a Liebenthal horse stallion. Figure S5. Plot of the mean Ln P(K) with the corresponding standard deviation (Stdev) from 10 repetitions of the STRUCTURE runs for K = 1 to 20. The maximum of Ln P(K) is indicated by vertical red dashed lines and the standard deviations for each K by gray vertical lines. Figure S6. Plots of the postprocessing results from CLUMPAK based on 10 independent runs of STRUCTURE for K = 1 to 20. Shown are the major modes for K = 2 to 18. Table S1. Sample number (N), distribution by sex and origin of the primitive horse populations. Table S2. List of microsatellite markers used in the study. Table S3. Test results of the marker LEX073 for Hardy-Weinberg-Equilibrium in 18 horse populations. Table S4. Characteristics of the 29 microsatellite markers analyzed in 688 horses from 18 horse populations. Size range of alleles (bp), number of alleles (N_A_), observed heterozygosity (H_o_), Wright’s F_ST_, and the coefficient of gene differentiation G_ST_ are given. Table S5. Population differentiation using F_ST_ estimates among the 18 horse populations based on 29 microsatellite markers. Table S6. Genetic distances among 18 horse populations using Nei’s standard genetic distance (D_s_) based on 29 microsatellite markers. Table S7. Genetic distances among 18 horse populations using Cavalli-Sforza chord distance (D_C_) based on 29 microsatellite markers. Table S8. Results from the analysis of 10 independent runs of STRUCTURE [30,31,32,33] with STRUCTURESELECTOR [34] to obtain the mean of the posterior probability ln P(G|K) (Ln P(K)) with its standard deviation (Stdev Ln P(K)), Ln′(K), |Ln″(K)| and ΔK (Delta K) for K = 1–20. The maximum of the mean Ln P(K) and ΔK are reached at K = 18 and this row is highlighted in yellow. Table S9. Membership coefficients from 10 independent STRUCTURE runs for 18 horse populations for K = 17. Table S10. Membership coefficients from 10 independent STRUCTURE runs for 18 horse populations for K = 18.
