# Supplementary material for: Genetic Diversity and Population Structure of Dülmen Wild, Liebenthal and Polish Konik Horses in Comparison with Przewalski, Sorraia, German Draught and Riding Horses
Source: Animals (Basel). 2024 Jul 31;14(15):2221. doi: 10.3390/ani14152221 (PMC11311111; doi:10.3390/ani14152221)
Supplement: Supplementary file 1 [file animals-14-02221-s001.zip › Supplementary Materials-Figure S1-S6-Table S1- Table S10.docx]

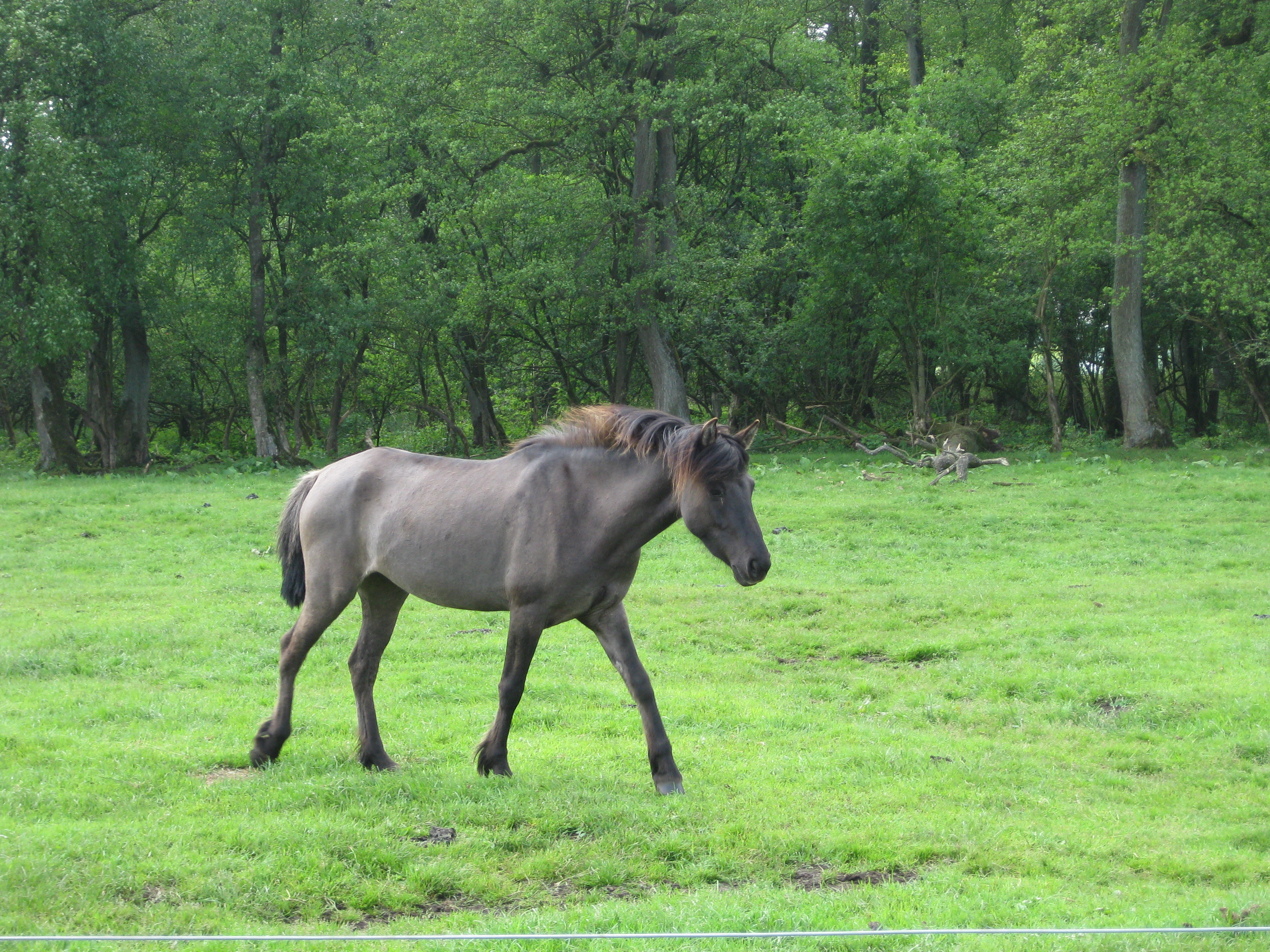


Figure S1. Photograph of a Dülmen wild horse mare in the Merfelder Bruch.


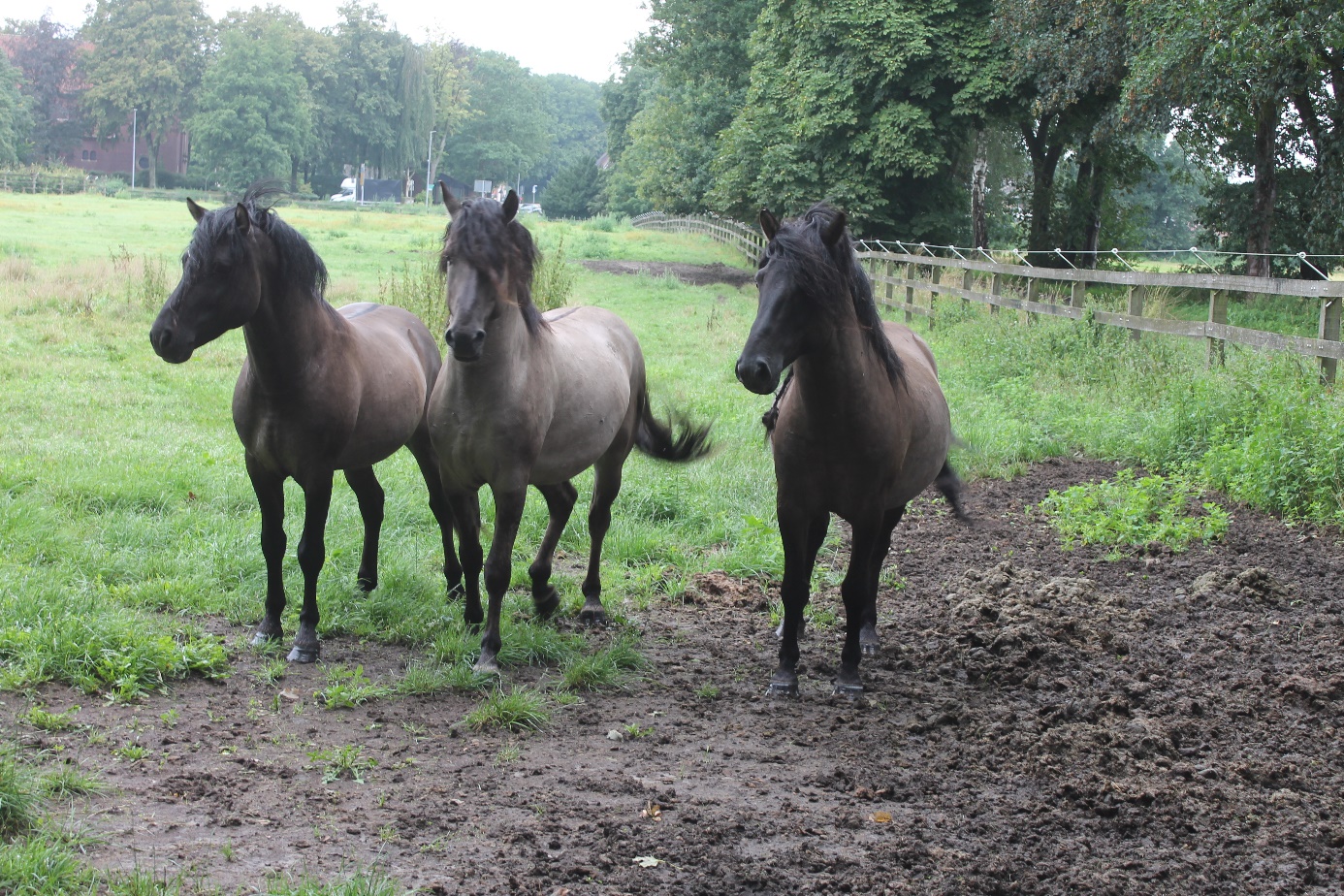


Figure S2. Photograph of Dülmen wild horse stallions born in the herd of the Merfelder Bruch.


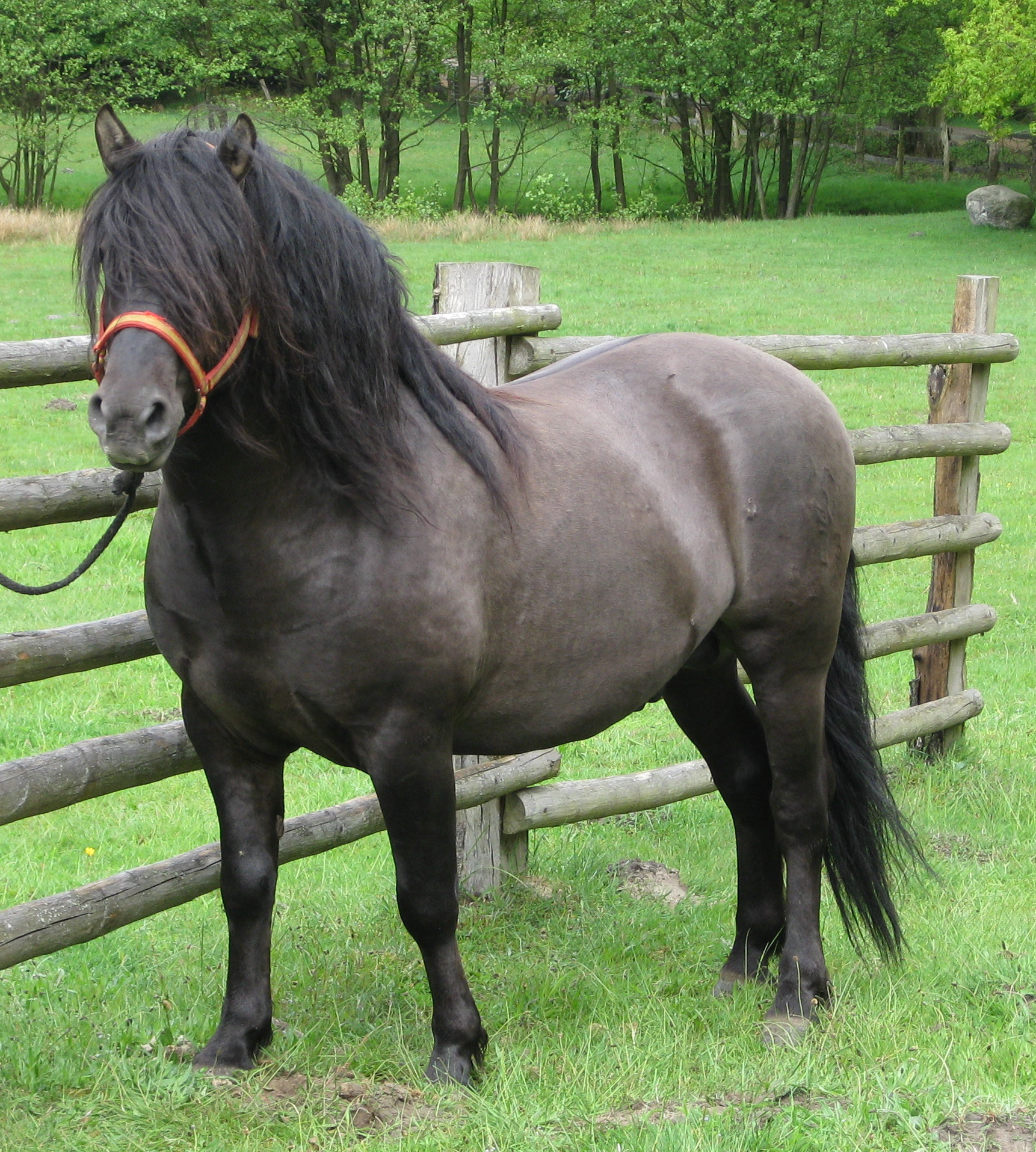


Figure S3. Photograph of a Dülmen horse gelding.


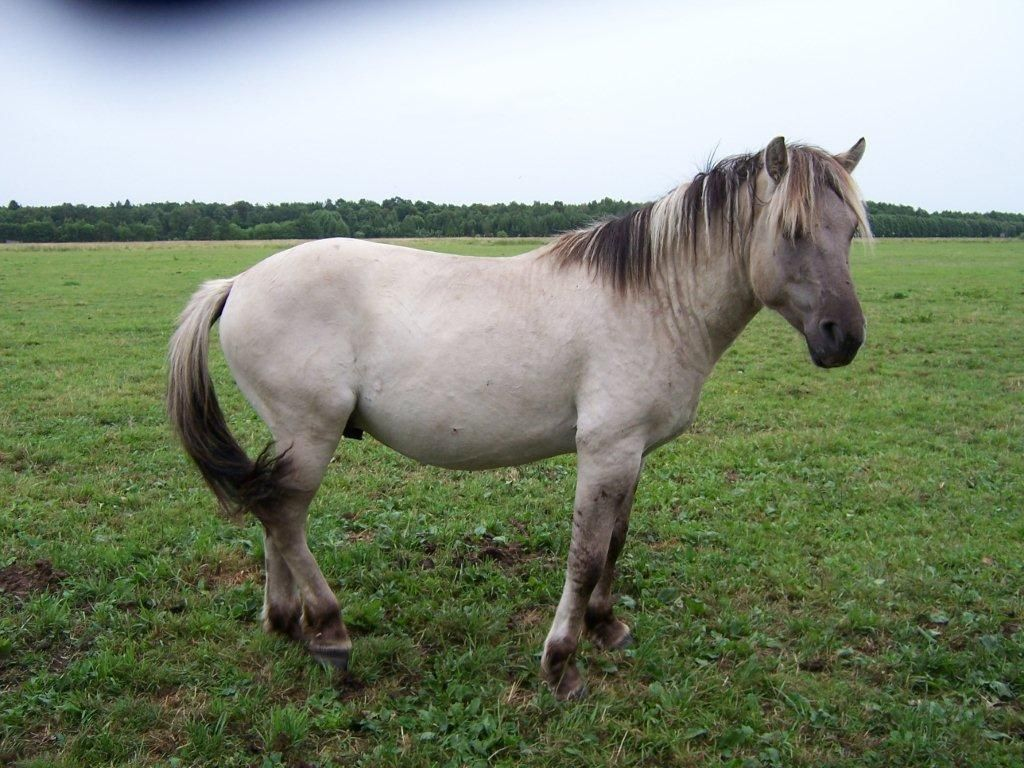


Figure S4. Photograph of a Liebenthal horse stallion.


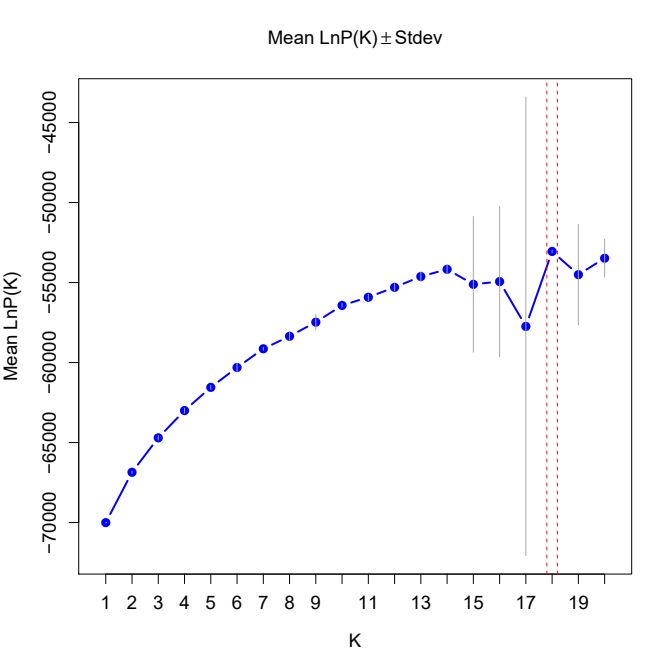


Figure S5. Plot of the mean Ln P(K) with the corresponding standard deviation (Stdev) from 10 repetitions of the STRUCTURE runs for K = 1 to 20. The maximum of Ln P(K) is indicated by vertical red dashed lines and the standard deviations for each K by gray vertical lines.


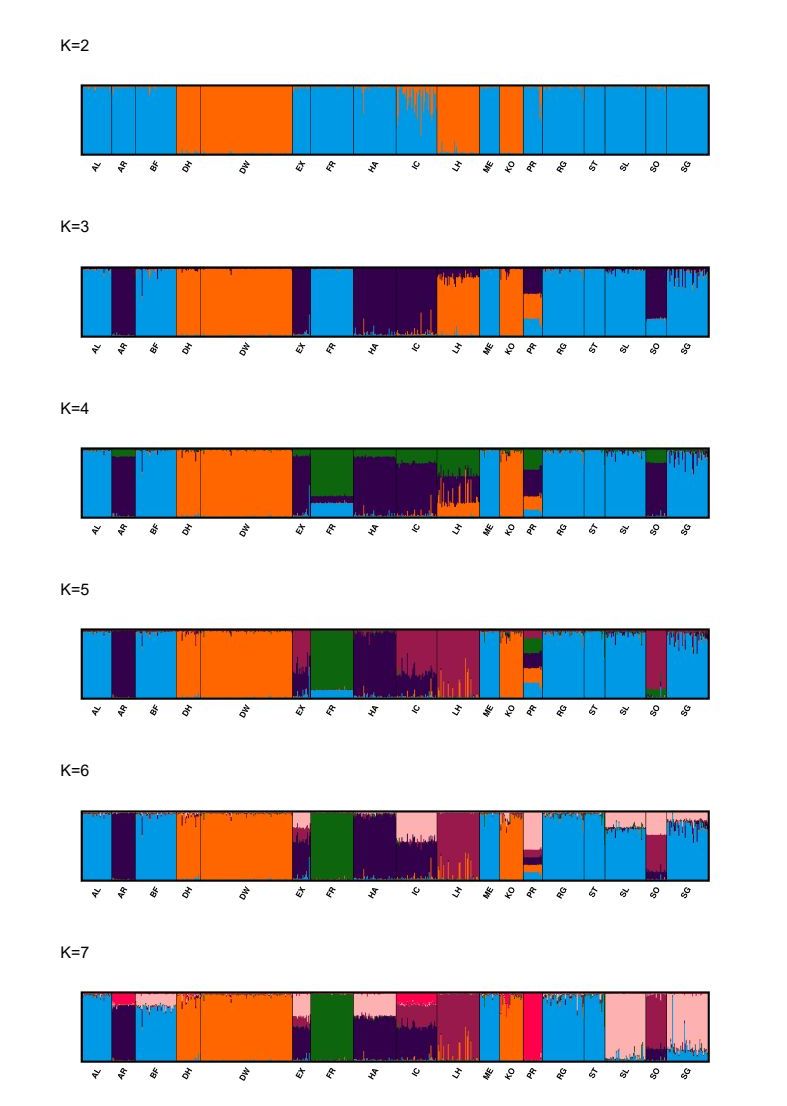


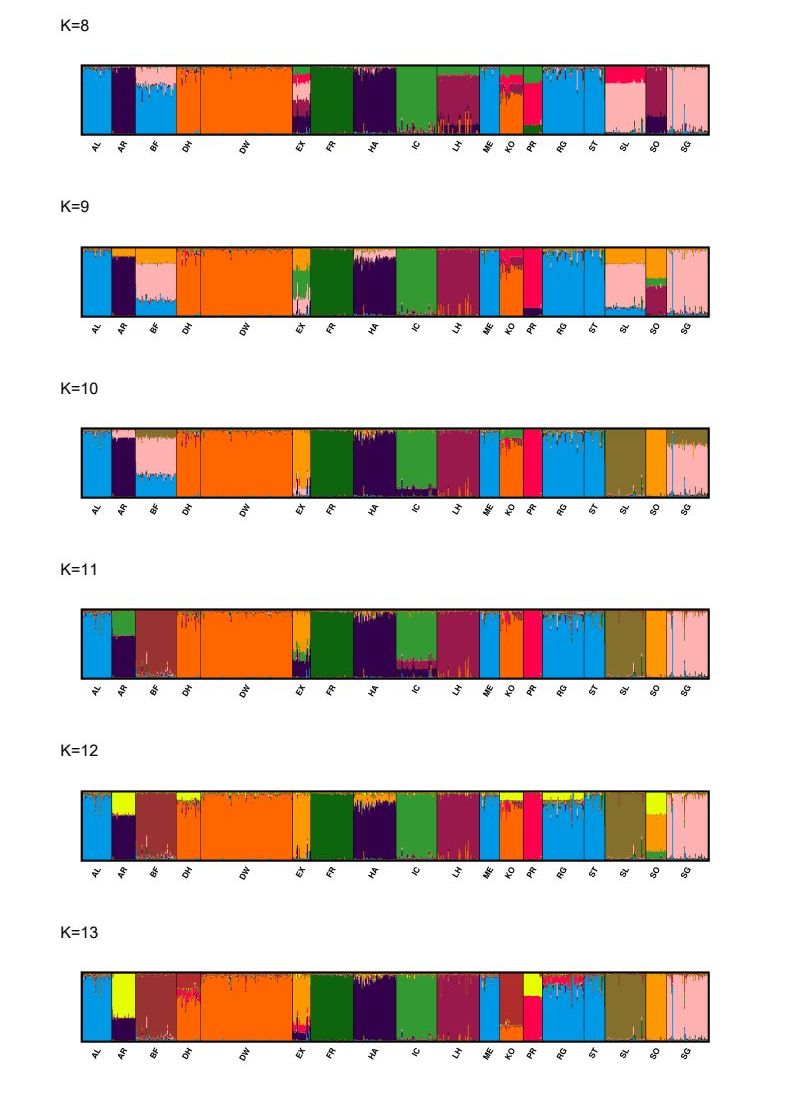


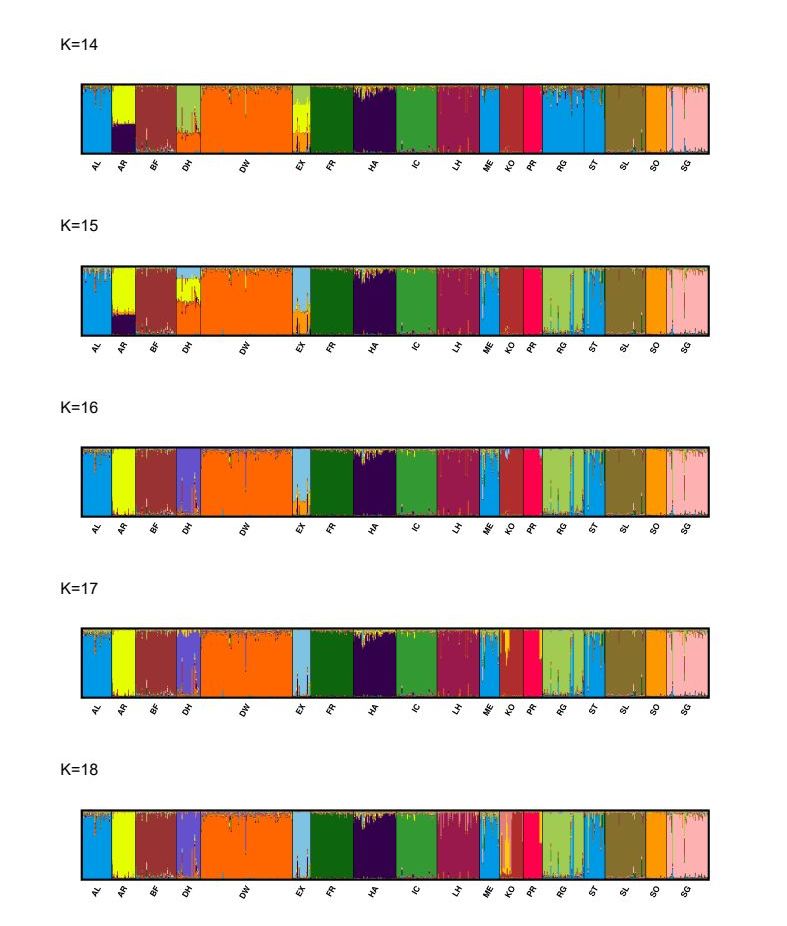


**Figure S6.** Plots of the postprocessing results from CLUMPAK based on 10 independent runs of STRUCTURE for K = 1 to 20. Shown are the major modes for K = 2 to 18.

**Table S1.** Sample number (N), distribution by sex and origin of the primitive horse populations.

| Population | N | Male | Female | Origin |  |
| --- | --- | --- | --- | --- | --- |
| Dülmen Horse | 27 | 7 | 20 | Stiftung Naturschutzpark Lüneburger Heide, Hof Tütsberg |  |
| Dülmen Wild Horse | 101 | 101 | 0 | Merfelder Bruch, Dülmen |  |
| Liebenthal Horse | 46 | 31 | 15 | Liebenthaler Pferdeherde e.V., Wildpferdegehege, Liebenwalde |  |
| Polish Konik Horse | 13 | 3 | 10 | Ilkerbruch, Wolfsburg |  |
|  | 13 | 7 | 6 | Allerniederung, Wolfsburg |  |

Table S2. List of microsatellite markers used in the study.

| Chromosome  location in  *Equus caballus* | Marker | Mean number  of alleles | Allele size (bp) |  | Optimized  annealing  temperature | Primer | IRD-  Fluorescent | Primer-  dilution | Accession-  number | Forward Primer Sequence  Reverse Primer Sequence | Reference |
| --- | --- | --- | --- | --- | --- | --- | --- | --- | --- | --- | --- |
|  |  |  | min. | max. | [°C] | [pmol] |  |  |  | 5´-3´ |  |
| ECA 01 | HMS007 | 5.050 | 170 | 182 | 58 | 4 | 700 | 01:10 | X74636 | CAGGAAACTCATGTTGATACCATC | Guerin, 1993 |
|  |  |  |  |  |  |  |  |  |  | TGTTGTTGAAACATACCTTGACTGT | |
| ECA 02 | ASB017 | 8.100 | 89 | 115 | 60 | 4 | 800 | 01:10 | [X93531](http://www.ncbi.nlm.nih.gov/entrez/viewer.fcgi?db=nucleotide&val=X93531) | GAGGGCGGTACCTTTGTACC | Breen et al. 1997 |
|  |  |  |  |  |  |  |  |  |  | ACCAGTCAGGATCTCCACCG |  |
| ECA 03 | LEX007 | 4.350 | 188 | 198 | 55 | 4 | 800 | 01:10 | [AF075610](http://www.ncbi.nlm.nih.gov/entrez/viewer.fcgi?db=nucleotide&val=AF075610) | GGTAGGGCTCTGGGATGA | Coogle et al. 1996c |
|  |  |  |  |  |  |  |  |  |  | AACACTGGGGAAAAGTCAG |  |
| ECA 04 | LEX033 | 6.350 | 178 | 204 | 55 | 6 | 800 | 01:10 | AF075635 | TTTAATCAAAGGATTCAGTTG | Coogle et al. 1996b |
|  |  |  |  |  |  |  |  |  |  | TTTCTCTTCAGGTGTCCTC |  |
| ECA 05 | LEX034 | 4.100 | 243 | 253 | 55 | 3 | 700 | 01:10 | AF075636 | GCGGAGGTAAGAAGTGGTAG | Coogle et al. 1997 |
|  |  |  |  |  |  |  |  |  |  | GGCCTAAGATGAGGGTGAA |  |
| ECA 06 | COR070 | 6.900 | 273 | 299 | 62 | 5 | 700 | 01:10 | [AF142607](http://www.ncbi.nlm.nih.gov/entrez/viewer.fcgi?db=nucleotide&val=AF142607) | CATCTGTTCCGTGGCATTA | Tallmadge et al. 1999b |
|  |  |  |  |  |  |  |  |  |  | TTCAGGTGTGGGTTTTGAATC |  |
| ECA 07 | SGCV28 | 4.050 | 149 | 165 | 62 | 3 | 800 | 01:10 | [U90604](http://www.ncbi.nlm.nih.gov/entrez/viewer.fcgi?db=nucleotide&val=U90604) | CTGTGGCAGCTGTCATCTTGG | Godard et al. 1997 |
|  |  |  |  |  |  |  |  |  |  | CCCAATTCCAGCCCAGCTTGC |  |
| ECA 08 | COR056 | 6.450 | 186 | 212 | 58 | 6 | 700 | 01:10 | [AF108373](http://www.ncbi.nlm.nih.gov/entrez/viewer.fcgi?db=nucleotide&val=AF108373) | AGATTCCAGGCATTAGGACC | Ruth et al. 1999 |
|  |  |  |  |  |  |  |  |  |  | TCAGGGACAATCTTCCTCAAG |  |
| ECA 10 | COR045 | 6.350 | 150 | 164 | 55 | 3 | 800 | 01:10 | AF108362 | TCTCTACCGCAAGTGAAACC | Hopman et al. 1999b |
|  |  |  |  |  |  |  |  |  |  | CTGAGCCCTTAACTTGTGGA |  |
| ECA 11 | LEX068 | 5.600 | 154 | 168 | 55 | 2 | 700 | 01:10 |  | AAATCCCGAGCTAAAATGTA | Coogle and Bailey 1999 |
|  |  |  |  |  |  |  |  |  |  | TAGGAAGATAGGATCACAAGG |  |
| ECA 12 | COR058 | 7.950 | 206 | 230 | 58 | 3 | 700 | 01:10 | AF108375 | GGGAAGGACGATGAGTGAC | Ruth et al. 1999 |
|  |  |  |  |  |  |  |  |  |  | CACCAGGCTAAGTAGCCAAAG |  |
| ECA 13 | COR069 | 5.600 | 259 | 283 | 58 | 4 | 700 | 01:10 | AF142606 | AGCCACCAGTCTGTTCTCTG | Tallmadge et al., 1999 |
|  |  |  |  |  |  |  |  |  |  | AATGTCCTTTGGTGGATGAAC |  |
| ECA 14 | VHL209 | 4.600 | 83 | 97 | 58 | 8 | 700 | 01:10 | Y08451 | TCTTACATCCTTCCATTACAACTA | van Haeringen et al. 1998 |
|  |  |  |  |  |  |  |  |  |  | TGATACATATGTACGTGAAAGGAT |  |
| ECA 15 | HTG006 | 3.900 | 82 | 104 | 55 | 3 | 700 | 01:10 | AF169167 | CCTGCTTGGAGGCTGTGATAAGAT | Ellegren et al. 1992 |
|  |  |  |  |  |  |  |  |  |  | GTTCACTGAATGTCAAATTCTGCT |  |
| ECA 16 | HTG003 | 4.550 | 114 | 124 | 55 | 4 | 800 | 01:10 | [AF169164](http://www.ncbi.nlm.nih.gov/entrez/viewer.fcgi?db=nucleotide&val=AF169164) | TAACCTGGGTGCAAAGCCACCCAT | Ellegren et al. 1992 |
|  |  |  |  |  |  |  |  |  |  | TCAGGGCCAATCTTCCTCAC |  |
| ECA 17 | COR007 | 5.250 | 153 | 175 | 58 | 2 | 800 | 01:10 | [AF083450](http://www.ncbi.nlm.nih.gov/entrez/viewer.fcgi?db=nucleotide&val=AF083450) | GTGTTGGATGAAGCGAATGA | Hopman et al. 1999 |
|  |  |  |  |  |  |  |  |  |  | GACTTGCCTGGCTTTGAGTC |  |
| ECA 18 | TKY019 | 5.400 | 144 | 160 | 55 | 3 | 700 | 01:10 | AB048330 | CTTCTGCTGATTCCTGAATG | Tozaki, 2000 |
|  |  |  |  |  |  |  |  |  |  | GGATCTCCTTAAATGGAACA |  |
| ECA 19 | LEX073 | 6.900 | 232 | 270 | 55 | 3 | 800 | 01:10 | [AF213359](http://www.ncbi.nlm.nih.gov/entrez/viewer.fcgi?db=nucleotide&val=AF213359) | CCAGCCATCCACTGGTAGAG | Bailey et al. 1999 |
|  |  |  |  |  |  |  |  |  |  | GGGAAAAGGGGAACCTTCTA |  |
| ECA 20 | UM011 | 7.000 | 160 | 180 | 58 | 10 | 800 | 01:10 | [AF195130](http://www.ncbi.nlm.nih.gov/entrez/viewer.fcgi?db=nucleotide&val=AF195130) | TGAAAGTAGAAAGGGATGTGG | Meyer et al. 1997 |
|  |  |  |  |  |  |  |  |  |  | TCTCAGAGCAGAAGTCCCTG |  |
| ECA 21 | SGCV16 | 4.800 | 140 | 188 | 55 | 6 | 800 | 01:10 | U90594 | AATTCTCAAATGGTTCAGTGA | Godard, 1997 |
|  |  |  |  |  |  |  |  |  |  | CTCCCTCCCTTCCTTCTA |  |
| ECA 22 | COR022 | 3.600 | 255 | 263 | 58 | 5 | 800 | 01:10 | [AF101391](http://www.ncbi.nlm.nih.gov/entrez/viewer.fcgi?db=nucleotide&val=AF101391) | AAGACGTGATGGGAAATCAA | Murphie et al. 1999 |
|  |  |  |  |  |  |  |  |  |  | AGAAAGTTTTCAAATGTGCCA |  |
| ECA 23 | LEX063 | 5.450 | 222 | 250 | 55 | 6 | 800 | 01:10 | [AF075663](http://www.ncbi.nlm.nih.gov/entrez/viewer.fcgi?db=nucleotide&val=AF075663) | CGGGGTGTGCATCTCTTAGG | Coogle and Bailey 1997 |
|  |  |  |  |  |  |  |  |  |  | TGGCGAATGCTGAATCTGG |  |
| ECA 24 | COR024 | 6.150 | 205 | 219 | 58 | 3 | 700 | 01:10 | [AF101393](http://www.ncbi.nlm.nih.gov/entrez/viewer.fcgi?db=nucleotide&val=AF101393) | CAAAAGTGATTGCCTTCGAT | Murphie et al. 1999 |
|  |  |  |  |  |  |  |  |  |  | TTGGAAGCTGGGTGATTG |  |
| ECA 25 | COR018 | 5.400 | 253 | 275 | 58 | 1 | 700 | 01:10 | [AF083461](http://www.ncbi.nlm.nih.gov/entrez/viewer.fcgi?db=nucleotide&val=AF083461) | AGTCTGGCAATATTGAGGATGT | Hopman et al. 1999 |
|  |  |  |  |  |  |  |  |  |  | AGCAGCTACCCTTTGAATACTG |  |
| ECA 26 | COR071 | 5.650 | 182 | 208 | 58 | 4 | 700 | 01:10 | [AF142608](http://www.ncbi.nlm.nih.gov/entrez/viewer.fcgi?db=nucleotide&val=AF142608) | CTTGGGCTACAACAGGGAATA | Tallmadge et al. 1999b |
|  |  |  |  |  |  |  |  |  |  | CTGCTATTTCAAACACTTGGA |  |
| ECA 27 | COR017 | 4.950 | 235 | 253 | 58 | 5 | 700 | 01:10 | [AF083460](http://www.ncbi.nlm.nih.gov/entrez/viewer.fcgi?db=nucleotide&val=AF083460) | GAAGGCCTGAAGCATTTACA | Hopman et al. 1999 |
|  |  |  |  |  |  |  |  |  |  | CGTAATGTTGACCAAACTTCA |  |
| ECA 28 | CA425 (UCDQ425) | 5.300 | 233 | 245 | 55 | 4 | 700 | 01:10 | [U67406](http://www.ncbi.nlm.nih.gov/entrez/viewer.fcgi?db=nucleotide&val=U67406) | AGCTGCCTCGTTAATTCA | Eggelston-Stott et al. 1997 |
|  |  |  |  |  |  |  |  |  |  | CTCATGTCCGCTTGTCTC |  |
| ECA 29 | COR082 | 5.400 | 198 | 228 | 58 | 5 | 800 | 01:10 | [AF154935](http://www.ncbi.nlm.nih.gov/entrez/viewer.fcgi?db=nucleotide&val=AF154935) | GCTTTTGTTTCTCAATCCTAGC | Tallmadge et al. 1999 |
|  |  |  |  |  |  |  |  |  |  | TGAAGTCAAATCCCTGCTTC |  |
| ECA 30 | VHL020 | 6.500 | 88 | 106 | 58 | 10 | 700 | 01:10 | [Y08451](http://www.ncbi.nlm.nih.gov/entrez/viewer.fcgi?db=nucleotide&val=Y08451) | TCTTACATCCTTCCATTACAACTA | van Haeringen et al. 1998 |
|  |  |  |  |  |  |  |  |  |  | TGATACATATGTACGTGAAAGGAT |  |
| ECA 31 | AHT034 | 5.550 | 121 | 141 | 60 | 5 | 700 | 01:10 | [AJ271519](http://www.ncbi.nlm.nih.gov/entrez/viewer.fcgi?db=nucleotide&val=AJ271519) | CTCAGGGCGAATGTTCCTC | Swinburne et al. 2000b |
|  |  |  |  |  |  |  |  |  |  | CCCCACCATGAGTCAAAAAC |  |

Marker HMS003 from Aberle et al. 2004 [18] was not used due to low heterozygosity and technical properties in amplification. All other 30 markers are identical to the marker set of Aberle et al. 2004 [18].

Table S3. Test results of the marker LEX073 for Hardy-Weinberg-Equilibrium in 18 horse populations.

| Population | DF | χ^2^ | *p*-Value |
| --- | --- | --- | --- |
| Altmaerkisch Coldblood | 21 | 21.421 | 0.433 |
| Arabian | 10 | 5.125 | 0.883 |
| Black Forest Horse | 28 | 14.424 | 0.984 |
| Dülmen Horse | 15 | 16.678 | 0.338 |
| Dülmen Wild Horse | 55 | 147.598 | 0.0001 |
| Exmoor Pony | 10 | 4.132 | 0.941 |
| Friesian | 10 | 6.006 | 0.815 |
| Hanoverian Warmblood | 21 | 22.002 | 0.399 |
| Icelandic Horse | 15 | 14.052 | 0.522 |
| Liebenthal Horse | 10 | 26.794 | 0.003 |
| Mecklenburg Coldblood | 21 | 17.0791 | 0.706 |
| Polish Konik Horse | 15 | 29.970 | 0.012 |
| Przewalski´s Horse | 15 | 25.210 | 0.047 |
| Rhenish German Coldblood | 10 | 5.737 | 0.837 |
| Saxon-Thuringa Coldblood | 15 | 20.232 | 0.163 |
| Schleswig Draught Horse | 21 | 20.540 | 0.487 |
| Sorraia Horse | 6 | 7.347 | 0.290 |
| South German Coldblood | 21 | 14.241 | 0.859 |

LEX073 was removed from further analyses due to significant deviations from Hardy-Weinberg-Equilibrium (HWE) in more than one population.

Table S4. Characteristics of the 29 microsatellite markers analysed in 688 horses from 18 horse populations. Size range of alleles (bp), number of alleles (N_A_), averages for the observed heterozygosity (H_O_), Wright’s F_ST_, coefficient of gene differentiation G_ST_, polymorphism information content (PIC) and allelic diversity (AD) are given.

| Locus | Range | N_A_ | H_O_ | F_ST_ | G_ST_ | PIC | AD |
| --- | --- | --- | --- | --- | --- | --- | --- |
| AHT034 | 121-141 | 9 | 0.709 | 0.172 | 0.160 | 0.802 | 0.825 |
| ASB017 | 89-131 | 19 | 0.762 | 0.165 | 0.153 | 0.890 | 0.898 |
| COR007 | 147-179 | 11 | 0.632 | 0.189 | 0.176 | 0.770 | 0.797 |
| COR017 | 235-255 | 9 | 0.627 | 0.164 | 0.152 | 0.666 | 0.703 |
| COR018 | 253-279 | 11 | 0.651 | 0.161 | 0.148 | 0.724 | 0.747 |
| COR022 | 253-263 | 4 | 0.563 | 0.192 | 0.180 | 0.613 | 0.663 |
| COR024 | 205-219 | 8 | 0.711 | 0.165 | 0.153 | 0.807 | 0.829 |
| COR045 | 211-229 | 10 | 0.723 | 0.143 | 0.130 | 0.808 | 0.828 |
| COR056 | 180-212 | 15 | 0.592 | 0.249 | 0.237 | 0.808 | 0.828 |
| COR058 | 206-232 | 14 | 0.786 | 0.173 | 0.160 | 0.901 | 0.908 |
| COR069 | 259-283 | 11 | 0.729 | 0.153 | 0.140 | 0.799 | 0.822 |
| COR070 | 263-299 | 13 | 0.706 | 0.149 | 0.136 | 0.809 | 0.829 |
| COR071 | 180-208 | 12 | 0.630 | 0.187 | 0.175 | 0.724 | 0.756 |
| COR082 | 198-232 | 10 | 0.647 | 0.173 | 0.161 | 0.747 | 0.776 |
| HMS007 | 170-184 | 8 | 0.626 | 0.195 | 0.182 | 0.735 | 0.770 |
| HTG003 | 112-128 | 8 | 0.540 | 0.201 | 0.189 | 0.652 | 0.682 |
| HTG006 | 82-104 | 9 | 0.360 | 0.232 | 0.220 | 0.416 | 0.435 |
| LEX007 | 186-202 | 9 | 0.609 | 0.173 | 0.160 | 0.706 | 0.745 |
| LEX033 | 178-204 | 11 | 0.695 | 0.190 | 0.177 | 0.816 | 0.835 |
| LEX034 | 239-253 | 7 | 0.592 | 0.158 | 0.146 | 0.595 | 0.656 |
| LEX063 | 222-250 | 10 | 0.652 | 0.226 | 0.214 | 0.754 | 0.785 |
| LEX068 | 144-170 | 10 | 0.658 | 0.197 | 0.184 | 0.827 | 0.846 |
| SGCV16 | 140-196 | 12 | 0.608 | 0.181 | 0.167 | 0.717 | 0.746 |
| SGCV28 | 149-165 | 7 | 0.553 | 0.193 | 0.179 | 0.662 | 0.697 |
| TKY019 | 144-164 | 8 | 0.696 | 0.169 | 0.157 | 0.783 | 0.808 |
| CA425 (UCDEQ425) | 227-247 | 10 | 0.573 | 0.206 | 0.193 | 0.662 | 0.699 |
| UM011 | 160-182 | 16 | 0.729 | 0.146 | 0.133 | 0.784 | 0.802 |
| VHL020 | 88-106 | 10 | 0.731 | 0.203 | 0.191 | 0.848 | 0.863 |
| VHL209 | 81-101 | 9 | 0.691 | 0.189 | 0.177 | 0.760 | 0.825 |
| All |  | 9.77 | 0.648 | 0.182 | 0.169 | 0.744 | 0.771 |

Table S5. Population differentiation using F_ST_ estimates among the 18 horse populations based on 29 microsatellite markers.

| Population | AL | AR | BF | DH | DW | EX | FR | HA | IC | LH | ME | KO | PR | RG | ST | SL | SO | SG |
| --- | --- | --- | --- | --- | --- | --- | --- | --- | --- | --- | --- | --- | --- | --- | --- | --- | --- | --- |
| Altmaerkisch Coldblood | 0.000 |  |  |  |  |  |  |  |  |  |  |  |  |  |  |  |  |  |
| Arabian | 0.133 | 0.000 |  |  |  |  |  |  |  |  |  |  |  |  |  |  |  |  |
| Black Forest Horse | 0.044 | 0.128 | 0.000 |  |  |  |  |  |  |  |  |  |  |  |  |  |  |  |
| Dülmen Horse | 0.076 | 0.124 | 0.080 | 0.000 |  |  |  |  |  |  |  |  |  |  |  |  |  |  |
| Dülmen Wild Horse | 0.066 | 0.126 | 0.080 | 0.048 | 0.000 |  |  |  |  |  |  |  |  |  |  |  |  |  |
| Exmoor Pony | 0.110 | 0.142 | 0.108 | 0.119 | 0.107 | 0.000 |  |  |  |  |  |  |  |  |  |  |  |  |
| Friesian | 0.104 | 0.184 | 0.125 | 0.152 | 0.133 | 0.179 | 0.000 |  |  |  |  |  |  |  |  |  |  |  |
| Hanoverian Warmblood | 0.076 | 0.090 | 0.075 | 0.073 | 0.077 | 0.099 | 0.117 | 0.000 |  |  |  |  |  |  |  |  |  |  |
| Icelandic Horse | 0.072 | 0.113 | 0.082 | 0.063 | 0.067 | 0.106 | 0.117 | 0.061 | 0.000 |  |  |  |  |  |  |  |  |  |
| Liebenthal Horse | 0.087 | 0.139 | 0.099 | 0.079 | 0.082 | 0.129 | 0.125 | 0.079 | 0.072 | 0.000 |  |  |  |  |  |  |  |  |
| Mecklenburg Coldblood | 0.022 | 0.134 | 0.053 | 0.079 | 0.065 | 0.112 | 0.109 | 0.082 | 0.077 | 0.089 | 0.000 |  |  |  |  |  |  |  |
| Polish Konik Horse | 0.124 | 0.160 | 0.125 | 0.105 | 0.076 | 0.163 | 0.149 | 0.111 | 0.095 | 0.109 | 0.119 | 0.000 |  |  |  |  |  |  |
| Przewalski´s Horse | 0.169 | 0.187 | 0.149 | 0.155 | 0.167 | 0.207 | 0.205 | 0.155 | 0.140 | 0.174 | 0.161 | 0.180 | 0.000 |  |  |  |  |  |
| Rhenish German Coldblood | 0.029 | 0.129 | 0.049 | 0.072 | 0.062 | 0.111 | 0.097 | 0.072 | 0.066 | 0.077 | 0.025 | 0.114 | 0.151 | 0.000 |  |  |  |  |
| Saxon-Thuringa Coldblood | 0.023 | 0.139 | 0.055 | 0.082 | 0.075 | 0.115 | 0.102 | 0.085 | 0.081 | 0.092 | 0.017 | 0.133 | 0.170 | 0.026 | 0.000 |  |  |  |
| Schleswig Draught Horse | 0.055 | 0.116 | 0.062 | 0.077 | 0.081 | 0.100 | 0.096 | 0.064 | 0.064 | 0.083 | 0.056 | 0.124 | 0.153 | 0.048 | 0.047 | 0.000 |  |  |
| Sorraia Horse | 0.143 | 0.210 | 0.145 | 0.168 | 0.158 | 0.162 | 0.204 | 0.126 | 0.146 | 0.138 | 0.148 | 0.208 | 0.256 | 0.137 | 0.139 | 0.130 | 0.000 |  |
| South German Coldblood | 0.052 | 0.111 | 0.046 | 0.074 | 0.063 | 0.093 | 0.098 | 0.060 | 0.058 | 0.071 | 0.052 | 0.104 | 0.134 | 0.044 | 0.057 | 0.048 | 0.132 | 0.000 |

Table S6. Genetic distances among 18 horse populations using Nei´s standard genetic distance (D_s_) based on 29 microsatellite markers.

| Population | AL | AR | BF | DH | DW | EX | FR | HA | IC | LH | ME | KO | PR | RG | ST | SL | SO | SG |
| --- | --- | --- | --- | --- | --- | --- | --- | --- | --- | --- | --- | --- | --- | --- | --- | --- | --- | --- |
| Altmaerkisch Coldblood | 0.000 |  |  |  |  |  |  |  |  |  |  |  |  |  |  |  |  |  |
| Arabian | 0.366 | 0.000 |  |  |  |  |  |  |  |  |  |  |  |  |  |  |  |  |
| Black Forest Horse | 0.165 | 0.332 | 0.000 |  |  |  |  |  |  |  |  |  |  |  |  |  |  |  |
| Dülmen Horse | 0.277 | 0.374 | 0.270 | 0.000 |  |  |  |  |  |  |  |  |  |  |  |  |  |  |
| Dülmen Wild Horse | 0.221 | 0.368 | 0.251 | 0.110 | 0.000 |  |  |  |  |  |  |  |  |  |  |  |  |  |
| Exmoor Pony | 0.316 | 0.374 | 0.312 | 0.373 | 0.307 | 0.000 |  |  |  |  |  |  |  |  |  |  |  |  |
| Friesian | 0.288 | 0.430 | 0.308 | 0.404 | 0.318 | 0.419 | 0.000 |  |  |  |  |  |  |  |  |  |  |  |
| Hanoverian Warmblood | 0.251 | 0.243 | 0.235 | 0.282 | 0.263 | 0.275 | 0.312 | 0.000 |  |  |  |  |  |  |  |  |  |  |
| Icelandic Horse | 0.258 | 0.340 | 0.285 | 0.256 | 0.260 | 0.310 | 0.378 | 0.235 | 0.000 |  |  |  |  |  |  |  |  |  |
| Liebenthal Horse | 0.299 | 0.426 | 0.307 | 0.270 | 0.257 | 0.395 | 0.342 | 0.295 | 0.270 | 0.000 |  |  |  |  |  |  |  |  |
| Mecklenburg Coldblood | 0.086 | 0.365 | 0.168 | 0.270 | 0.238 | 0.323 | 0.285 | 0.273 | 0.271 | 0.305 | 0.000 |  |  |  |  |  |  |  |
| Polish Konik Horse | 0.366 | 0.434 | 0.359 | 0.282 | 0.226 | 0.439 | 0.402 | 0.355 | 0.325 | 0.304 | 0.349 | 0.000 |  |  |  |  |  |  |
| Przewalski´s Horse | 0.516 | 0.548 | 0.463 | 0.489 | 0.491 | 0.569 | 0.553 | 0.515 | 0.473 | 0.518 | 0.484 | 0.500 | 0.000 |  |  |  |  |  |
| Rhenish German Coldblood | 0.106 | 0.347 | 0.146 | 0.244 | 0.216 | 0.295 | 0.256 | 0.241 | 0.240 | 0.290 | 0.091 | 0.355 | 0.493 | 0.000 |  |  |  |  |
| Saxon-Thuringa Coldblood | 0.087 | 0.380 | 0.169 | 0.283 | 0.249 | 0.330 | 0.264 | 0.280 | 0.295 | 0.327 | 0.063 | 0.398 | 0.522 | 0.090 | 0.000 |  |  |  |
| Schleswig Draught Horse | 0.215 | 0.327 | 0.182 | 0.281 | 0.274 | 0.298 | 0.273 | 0.217 | 0.256 | 0.324 | 0.190 | 0.385 | 0.487 | 0.175 | 0.180 | 0.000 |  |  |
| Sorraia Horse | 0.372 | 0.481 | 0.391 | 0.471 | 0.425 | 0.339 | 0.462 | 0.366 | 0.392 | 0.404 | 0.373 | 0.508 | 0.681 | 0.380 | 0.364 | 0.351 | 0.000 |  |
| South German Coldblood | 0.192 | 0.308 | 0.149 | 0.279 | 0.231 | 0.266 | 0.259 | 0.200 | 0.235 | 0.281 | 0.170 | 0.333 | 0.439 | 0.144 | 0.188 | 0.165 | 0.347 | 0.000 |

Table S7. Genetic distances among 18 horse populations using Cavalli-Sforza chord distance (D_C_) based on 29 microsatellite markers.

| Population | AL | AR | BF | DH | DW | EX | FR | HA | IC | LH | ME | KO | PR | RG | ST | SL | SO | SG |
| --- | --- | --- | --- | --- | --- | --- | --- | --- | --- | --- | --- | --- | --- | --- | --- | --- | --- | --- |
| Altmaerkisch Coldblood | 0.000 |  |  |  |  |  |  |  |  |  |  |  |  |  |  |  |  |  |
| Arabian | 0.522 | 0.000 |  |  |  |  |  |  |  |  |  |  |  |  |  |  |  |  |
| Black Forest Horse | 0.343 | 0.492 | 0.000 |  |  |  |  |  |  |  |  |  |  |  |  |  |  |  |
| Dülmen Horse | 0.450 | 0.516 | 0.443 | 0.000 |  |  |  |  |  |  |  |  |  |  |  |  |  |  |
| Dülmen Wild Horse | 0.399 | 0.512 | 0.427 | 0.287 | 0.000 |  |  |  |  |  |  |  |  |  |  |  |  |  |
| Exmoor Pony | 0.487 | 0.528 | 0.474 | 0.525 | 0.478 | 0.000 |  |  |  |  |  |  |  |  |  |  |  |  |
| Friesian | 0.451 | 0.559 | 0.478 | 0.552 | 0.480 | 0.562 | 0.000 |  |  |  |  |  |  |  |  |  |  |  |
| Hanoverian Warmblood | 0.441 | 0.427 | 0.420 | 0.454 | 0.442 | 0.463 | 0.484 | 0.000 |  |  |  |  |  |  |  |  |  |  |
| Icelandic Horse | 0.431 | 0.508 | 0.464 | 0.440 | 0.436 | 0.489 | 0.539 | 0.422 | 0.000 |  |  |  |  |  |  |  |  |  |
| Liebenthal Horse | 0.470 | 0.564 | 0.471 | 0.453 | 0.443 | 0.537 | 0.511 | 0.470 | 0.446 | 0.000 |  |  |  |  |  |  |  |  |
| Mecklenburg Coldblood | 0.239 | 0.521 | 0.348 | 0.442 | 0.408 | 0.493 | 0.459 | 0.455 | 0.446 | 0.475 | 0.000 |  |  |  |  |  |  |  |
| Polish Konik Horse | 0.521 | 0.560 | 0.513 | 0.464 | 0.407 | 0.578 | 0.536 | 0.518 | 0.496 | 0.481 | 0.506 | 0.000 |  |  |  |  |  |  |
| Przewalski´s Horse | 0.630 | 0.647 | 0.596 | 0.614 | 0.612 | 0.666 | 0.651 | 0.633 | 0.603 | 0.636 | 0.612 | 0.613 | 0.000 |  |  |  |  |  |
| Rhenish German Coldblood | 0.267 | 0.511 | 0.331 | 0.422 | 0.392 | 0.477 | 0.438 | 0.429 | 0.420 | 0.464 | 0.255 | 0.515 | 0.614 | 0.000 |  |  |  |  |
| Saxon-Thuringa Coldblood | 0.246 | 0.535 | 0.352 | 0.459 | 0.428 | 0.497 | 0.444 | 0.468 | 0.471 | 0.494 | 0.207 | 0.547 | 0.635 | 0.260 | 0.000 |  |  |  |
| Schleswig Draught Horse | 0.396 | 0.496 | 0.369 | 0.455 | 0.449 | 0.463 | 0.447 | 0.409 | 0.437 | 0.488 | 0.376 | 0.536 | 0.613 | 0.364 | 0.364 | 0.000 |  |  |
| Sorraia Horse | 0.534 | 0.611 | 0.548 | 0.603 | 0.571 | 0.507 | 0.593 | 0.534 | 0.552 | 0.550 | 0.539 | 0.621 | 0.728 | 0.543 | 0.525 | 0.516 | 0.000 |  |
| South German Coldblood | 0.378 | 0.483 | 0.332 | 0.454 | 0.399 | 0.451 | 0.436 | 0.395 | 0.423 | 0.458 | 0.355 | 0.499 | 0.581 | 0.330 | 0.380 | 0.357 | 0.518 | 0.000 |

**Table S8.** Results from the analysis of 10 independent runs of STRUCTURE [30-33] with STRUCTURESELECTOR [34] to obtain the mean of the posterior probability ln P(G|K) (Ln P(K)) with its standard deviation (Stdev Ln P(K)), Ln’(K), |Ln’’(K)| and ΔK (Delta K) for K = 1-20. The maximum of the mean Ln P(K) and ΔK are reached at K=18 and this row is highlighted in yellow.


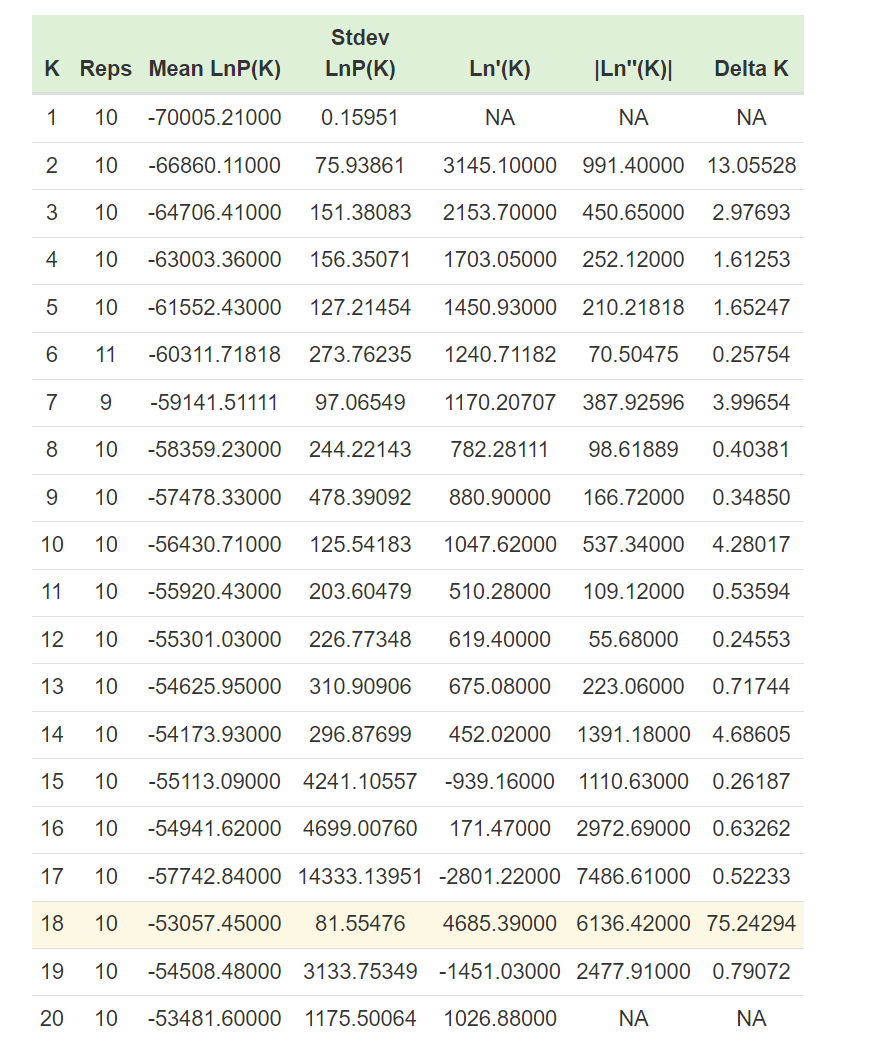


NA: not applicable.
