# Supplementary material for: Genetic Diversity and Population Structure of Dülmen Wild, Liebenthal and Polish Konik Horses in Comparison with Przewalski, Sorraia, German Draught and Riding Horses
Source: Animals (Basel). 2024 Jul 31;14(15):2221. doi: 10.3390/ani14152221 (PMC11311111; doi:10.3390/ani14152221)
Supplement: Supplementary file 1 [file animals-14-02221-s001.zip › Supplementary-Table-S09-K17-membership-coefficients.docx]

**Table S9.** Membership coefficients from 10 independent STRUCTURE runs for 18 horse populations for K = 17.

| **Population** | **Abbreviation** | **Running number** |
| --- | --- | --- |
| Altmaerkisch Coldblood | AL | 1 |
| Arabian | AR | 2 |
| Black Forest Horse | BF | 3 |
| Dülmen Horse | DH | 4 |
| Dülmen Wild Horse | DW | 5 |
| Exmoor Pony | EX | 6 |
| Friesian | FR | 7 |
| Hanoverian Warmblood | HA | 8 |
| Icelandic Horse | IC | 9 |
| Liebenthal Horse | LH | 10 |
| Mecklenburg Coldblood | ME | 11 |
| Polish Konik Horse | KO | 12 |
| Przewalski Horse | PR | 13 |
| Rhenish German Coldblood | RG | 14 |
| Saxon-Thuringa Coldblood | ST | 15 |
| Schleswig Draught Horse | SL | 16 |
| Sorraia Horse | SO | 17 |
| South German Coldblood | SG | 18 |

Run 1: Given Inferred Clusters Number of

Pop 1 2 3 4 5 6 7 8 9 10 11 12 13 14 15 16 17 Individuals

1: 0.014 0.005 0.002 0.004 0.005 0.903 0.004 0.003 0.010 0.005 0.005 0.014 0.005 0.004 0.009 0.004 0.005 32

2: 0.002 0.003 0.005 0.002 0.014 0.003 0.003 0.943 0.003 0.005 0.002 0.003 0.002 0.003 0.002 0.003 0.002 26

3: 0.011 0.013 0.002 0.005 0.004 0.010 0.005 0.005 0.006 0.003 0.004 0.908 0.005 0.004 0.005 0.005 0.006 45

4: 0.005 0.004 0.002 0.006 0.006 0.006 0.019 0.004 0.005 0.002 0.004 0.004 0.008 0.003 0.056 0.862 0.003 27

5: 0.004 0.004 0.002 0.003 0.004 0.005 0.008 0.004 0.003 0.003 0.005 0.003 0.004 0.003 0.921 0.021 0.004 101

6: 0.007 0.006 0.006 0.885 0.030 0.011 0.002 0.016 0.005 0.008 0.004 0.005 0.002 0.002 0.004 0.003 0.005 20

7: 0.003 0.003 0.001 0.003 0.003 0.003 0.003 0.002 0.003 0.003 0.002 0.002 0.003 0.002 0.004 0.003 0.958 47

8: 0.006 0.010 0.002 0.011 0.901 0.005 0.004 0.011 0.007 0.007 0.009 0.006 0.005 0.003 0.004 0.005 0.005 47

9: 0.004 0.004 0.003 0.005 0.007 0.006 0.004 0.008 0.004 0.005 0.921 0.004 0.006 0.004 0.005 0.006 0.003 45

10: 0.003 0.002 0.002 0.002 0.003 0.003 0.036 0.002 0.002 0.003 0.003 0.002 0.899 0.014 0.016 0.004 0.003 47

11: 0.080 0.019 0.002 0.004 0.003 0.804 0.004 0.006 0.017 0.004 0.007 0.018 0.003 0.005 0.005 0.005 0.013 22

12: 0.002 0.002 0.002 0.002 0.001 0.001 0.884 0.001 0.002 0.002 0.002 0.002 0.002 0.087 0.005 0.003 0.002 26

13: 0.001 0.002 0.915 0.002 0.001 0.001 0.004 0.001 0.001 0.001 0.002 0.001 0.002 0.060 0.002 0.002 0.001 21

14: 0.824 0.008 0.002 0.006 0.004 0.081 0.009 0.004 0.010 0.005 0.009 0.010 0.005 0.004 0.005 0.005 0.010 46

15: 0.071 0.005 0.002 0.003 0.002 0.803 0.004 0.002 0.038 0.006 0.002 0.009 0.003 0.003 0.003 0.003 0.041 23

16: 0.008 0.008 0.002 0.003 0.004 0.006 0.003 0.004 0.918 0.003 0.003 0.015 0.002 0.003 0.004 0.004 0.012 45

17: 0.002 0.003 0.001 0.006 0.003 0.002 0.002 0.003 0.002 0.963 0.002 0.002 0.002 0.001 0.002 0.002 0.002 23

18: 0.034 0.859 0.002 0.007 0.005 0.011 0.004 0.005 0.009 0.010 0.007 0.019 0.005 0.008 0.005 0.005 0.007 45

Run 2:

1: 0.010 0.005 0.004 0.014 0.005 0.903 0.004 0.005 0.005 0.002 0.014 0.004 0.005 0.005 0.003 0.004 0.009 32

2: 0.003 0.002 0.002 0.003 0.002 0.003 0.003 0.003 0.005 0.005 0.002 0.003 0.014 0.002 0.943 0.003 0.002 26

3: 0.006 0.006 0.005 0.909 0.005 0.010 0.004 0.013 0.003 0.002 0.011 0.005 0.004 0.004 0.005 0.005 0.005 45

4: 0.006 0.003 0.006 0.004 0.008 0.006 0.003 0.004 0.002 0.002 0.005 0.862 0.006 0.004 0.004 0.019 0.056 27

5: 0.003 0.004 0.003 0.003 0.005 0.005 0.002 0.004 0.003 0.002 0.004 0.021 0.004 0.005 0.004 0.008 0.922 101

6: 0.005 0.005 0.885 0.005 0.002 0.011 0.002 0.006 0.008 0.006 0.007 0.003 0.030 0.004 0.016 0.002 0.004 20

7: 0.003 0.958 0.003 0.002 0.003 0.003 0.002 0.003 0.003 0.001 0.003 0.003 0.003 0.002 0.002 0.003 0.004 47

8: 0.007 0.005 0.010 0.006 0.005 0.005 0.003 0.009 0.007 0.002 0.006 0.005 0.902 0.009 0.011 0.004 0.004 47

9: 0.004 0.003 0.005 0.004 0.006 0.006 0.004 0.004 0.005 0.003 0.004 0.005 0.007 0.922 0.008 0.004 0.005 45

10: 0.002 0.003 0.002 0.002 0.904 0.003 0.006 0.002 0.003 0.001 0.003 0.004 0.003 0.003 0.002 0.040 0.016 47

11: 0.017 0.013 0.004 0.018 0.003 0.803 0.005 0.018 0.004 0.002 0.082 0.005 0.003 0.007 0.006 0.004 0.004 22

12: 0.001 0.002 0.002 0.002 0.002 0.001 0.103 0.002 0.002 0.002 0.001 0.002 0.001 0.002 0.001 0.871 0.004 26

13: 0.001 0.001 0.001 0.001 0.002 0.001 0.072 0.002 0.001 0.905 0.001 0.002 0.001 0.002 0.001 0.003 0.001 21

14: 0.010 0.010 0.006 0.009 0.005 0.081 0.003 0.008 0.004 0.002 0.826 0.005 0.004 0.009 0.004 0.010 0.005 46

15: 0.037 0.042 0.003 0.009 0.003 0.802 0.002 0.005 0.007 0.002 0.072 0.003 0.002 0.002 0.002 0.004 0.003 23

16: 0.918 0.012 0.003 0.015 0.002 0.006 0.003 0.008 0.003 0.002 0.008 0.004 0.004 0.003 0.004 0.003 0.004 45

17: 0.002 0.002 0.006 0.002 0.002 0.002 0.001 0.003 0.963 0.001 0.002 0.002 0.003 0.002 0.003 0.002 0.002 23

18: 0.008 0.007 0.007 0.020 0.005 0.011 0.003 0.863 0.010 0.002 0.034 0.005 0.005 0.007 0.005 0.004 0.005 45

Run 3:

1: 0.003 0.010 0.005 0.009 0.014 0.005 0.005 0.002 0.004 0.005 0.004 0.004 0.005 0.005 0.004 0.014 0.903 32

2: 0.943 0.003 0.005 0.002 0.002 0.002 0.002 0.005 0.002 0.014 0.003 0.003 0.002 0.003 0.003 0.003 0.003 26

3: 0.005 0.006 0.003 0.005 0.011 0.006 0.004 0.002 0.004 0.004 0.005 0.005 0.005 0.013 0.004 0.909 0.010 45

4: 0.004 0.006 0.002 0.056 0.005 0.003 0.004 0.002 0.006 0.006 0.862 0.019 0.008 0.004 0.003 0.004 0.006 27

5: 0.004 0.003 0.003 0.922 0.004 0.004 0.005 0.002 0.003 0.004 0.021 0.008 0.004 0.004 0.002 0.003 0.005 101

6: 0.016 0.005 0.008 0.004 0.007 0.005 0.004 0.006 0.885 0.030 0.003 0.002 0.002 0.006 0.002 0.005 0.011 20

7: 0.002 0.003 0.003 0.004 0.003 0.958 0.002 0.001 0.003 0.003 0.003 0.003 0.003 0.003 0.002 0.002 0.003 47

8: 0.011 0.007 0.007 0.004 0.006 0.005 0.009 0.002 0.011 0.902 0.005 0.004 0.005 0.010 0.003 0.006 0.005 47

9: 0.008 0.004 0.005 0.005 0.004 0.003 0.922 0.003 0.005 0.007 0.006 0.004 0.006 0.004 0.004 0.004 0.006 45

10: 0.002 0.002 0.003 0.016 0.003 0.003 0.003 0.001 0.002 0.003 0.004 0.041 0.903 0.002 0.005 0.002 0.003 47

11: 0.006 0.017 0.004 0.005 0.083 0.013 0.006 0.002 0.004 0.003 0.005 0.004 0.003 0.018 0.005 0.019 0.801 22

12: 0.001 0.001 0.002 0.004 0.001 0.002 0.002 0.002 0.001 0.001 0.002 0.871 0.002 0.002 0.102 0.002 0.001 26

13: 0.001 0.001 0.001 0.001 0.001 0.001 0.002 0.905 0.001 0.001 0.002 0.003 0.002 0.002 0.072 0.001 0.001 21

14: 0.004 0.010 0.004 0.005 0.825 0.010 0.009 0.002 0.006 0.004 0.005 0.010 0.005 0.008 0.003 0.009 0.081 46

15: 0.002 0.038 0.007 0.003 0.073 0.042 0.002 0.002 0.003 0.002 0.003 0.004 0.003 0.005 0.002 0.009 0.800 23

16: 0.004 0.918 0.003 0.004 0.008 0.012 0.003 0.002 0.003 0.004 0.004 0.003 0.002 0.008 0.003 0.015 0.006 45

17: 0.003 0.002 0.963 0.002 0.002 0.002 0.002 0.001 0.006 0.003 0.002 0.002 0.002 0.003 0.001 0.002 0.002 23

18: 0.005 0.009 0.010 0.005 0.034 0.007 0.007 0.002 0.007 0.005 0.005 0.004 0.005 0.863 0.003 0.019 0.011 45

Run 4:

1: 0.002 0.005 0.903 0.014 0.003 0.004 0.005 0.004 0.005 0.004 0.005 0.014 0.005 0.004 0.009 0.009 0.005 32

2: 0.005 0.002 0.002 0.003 0.943 0.002 0.002 0.003 0.014 0.003 0.002 0.002 0.005 0.003 0.003 0.002 0.003 26

3: 0.002 0.004 0.010 0.909 0.005 0.004 0.006 0.004 0.004 0.005 0.005 0.011 0.003 0.005 0.006 0.005 0.013 45

4: 0.002 0.004 0.006 0.004 0.004 0.006 0.003 0.003 0.006 0.019 0.008 0.005 0.002 0.862 0.006 0.056 0.004 27

5: 0.002 0.005 0.005 0.003 0.004 0.003 0.004 0.002 0.004 0.008 0.004 0.004 0.003 0.021 0.003 0.922 0.004 101

6: 0.006 0.004 0.011 0.004 0.016 0.885 0.005 0.002 0.030 0.002 0.002 0.007 0.008 0.003 0.005 0.004 0.006 20

7: 0.001 0.002 0.003 0.002 0.002 0.003 0.958 0.002 0.003 0.003 0.003 0.003 0.003 0.003 0.003 0.004 0.003 47

8: 0.002 0.009 0.005 0.006 0.011 0.011 0.005 0.003 0.902 0.004 0.005 0.006 0.007 0.005 0.007 0.005 0.010 47

9: 0.003 0.921 0.006 0.004 0.008 0.005 0.003 0.004 0.007 0.004 0.006 0.004 0.005 0.006 0.004 0.005 0.004 45

10: 0.002 0.003 0.003 0.002 0.002 0.002 0.003 0.006 0.003 0.041 0.903 0.003 0.003 0.004 0.002 0.016 0.002 47

11: 0.002 0.007 0.803 0.019 0.006 0.004 0.013 0.005 0.003 0.004 0.003 0.082 0.004 0.005 0.017 0.005 0.018 22

12: 0.002 0.002 0.001 0.002 0.001 0.002 0.002 0.102 0.001 0.871 0.002 0.001 0.002 0.002 0.001 0.004 0.002 26

13: 0.905 0.002 0.001 0.001 0.001 0.001 0.001 0.072 0.001 0.003 0.002 0.001 0.001 0.002 0.001 0.001 0.002 21

14: 0.002 0.009 0.082 0.009 0.004 0.006 0.010 0.003 0.004 0.010 0.005 0.825 0.004 0.005 0.010 0.005 0.008 46

15: 0.002 0.002 0.802 0.009 0.002 0.003 0.042 0.002 0.002 0.004 0.003 0.072 0.007 0.003 0.038 0.003 0.005 23

16: 0.002 0.003 0.006 0.014 0.003 0.003 0.012 0.003 0.004 0.003 0.002 0.008 0.003 0.004 0.918 0.004 0.008 45

17: 0.001 0.002 0.002 0.002 0.003 0.006 0.002 0.001 0.003 0.002 0.002 0.002 0.963 0.002 0.002 0.002 0.003 23

18: 0.002 0.007 0.011 0.019 0.005 0.007 0.007 0.003 0.005 0.004 0.005 0.034 0.010 0.005 0.008 0.005 0.864 45

Run 5:

1: 0.002 0.009 0.004 0.005 0.014 0.003 0.004 0.005 0.005 0.005 0.005 0.014 0.004 0.903 0.005 0.004 0.010 32

2: 0.005 0.002 0.002 0.005 0.002 0.943 0.003 0.003 0.002 0.014 0.002 0.003 0.003 0.003 0.002 0.003 0.003 26

3: 0.002 0.005 0.005 0.003 0.011 0.005 0.005 0.013 0.005 0.004 0.006 0.909 0.004 0.010 0.004 0.005 0.006 45

4: 0.002 0.056 0.006 0.002 0.005 0.004 0.019 0.004 0.008 0.006 0.003 0.004 0.003 0.006 0.004 0.862 0.006 27

5: 0.002 0.921 0.003 0.003 0.004 0.004 0.008 0.004 0.004 0.004 0.004 0.003 0.002 0.005 0.005 0.022 0.003 101

6: 0.006 0.004 0.885 0.008 0.007 0.016 0.002 0.006 0.002 0.030 0.005 0.005 0.002 0.011 0.004 0.003 0.005 20

7: 0.001 0.004 0.003 0.003 0.003 0.002 0.003 0.003 0.003 0.003 0.958 0.002 0.002 0.003 0.002 0.003 0.003 47

8: 0.002 0.004 0.010 0.007 0.006 0.011 0.004 0.010 0.005 0.902 0.005 0.006 0.003 0.005 0.009 0.005 0.007 47

9: 0.003 0.005 0.005 0.005 0.004 0.008 0.004 0.004 0.006 0.007 0.003 0.004 0.004 0.006 0.922 0.006 0.004 45

10: 0.001 0.016 0.002 0.003 0.003 0.002 0.041 0.003 0.903 0.003 0.003 0.002 0.006 0.003 0.003 0.004 0.002 47

11: 0.002 0.005 0.004 0.004 0.082 0.006 0.004 0.018 0.003 0.003 0.013 0.018 0.005 0.802 0.007 0.005 0.017 22

12: 0.002 0.004 0.002 0.002 0.001 0.001 0.871 0.002 0.002 0.001 0.002 0.002 0.102 0.001 0.002 0.002 0.001 26

13: 0.905 0.001 0.001 0.001 0.001 0.001 0.003 0.002 0.002 0.001 0.001 0.001 0.072 0.001 0.002 0.002 0.001 21

14: 0.002 0.005 0.006 0.005 0.826 0.004 0.010 0.008 0.005 0.004 0.010 0.009 0.003 0.081 0.009 0.005 0.010 46

15: 0.002 0.003 0.003 0.007 0.072 0.002 0.004 0.005 0.003 0.002 0.042 0.009 0.002 0.801 0.002 0.003 0.038 23

16: 0.002 0.004 0.003 0.003 0.008 0.003 0.003 0.008 0.002 0.004 0.012 0.014 0.003 0.006 0.003 0.004 0.918 45

17: 0.001 0.002 0.006 0.963 0.002 0.003 0.002 0.003 0.002 0.003 0.002 0.002 0.001 0.002 0.002 0.002 0.002 23

18: 0.002 0.005 0.007 0.010 0.034 0.005 0.004 0.863 0.005 0.005 0.007 0.019 0.003 0.011 0.007 0.005 0.008 45

Run 6:

1: 0.014 0.005 0.903 0.014 0.010 0.005 0.005 0.004 0.009 0.004 0.005 0.004 0.005 0.002 0.005 0.004 0.003 32

2: 0.003 0.002 0.003 0.002 0.003 0.003 0.002 0.003 0.002 0.003 0.005 0.002 0.014 0.005 0.002 0.003 0.943 26

3: 0.909 0.005 0.010 0.011 0.006 0.013 0.006 0.005 0.005 0.005 0.003 0.005 0.004 0.002 0.004 0.004 0.005 45

4: 0.004 0.008 0.006 0.005 0.006 0.004 0.003 0.019 0.056 0.862 0.002 0.006 0.006 0.002 0.004 0.003 0.004 27

5: 0.003 0.004 0.005 0.004 0.003 0.004 0.004 0.008 0.922 0.021 0.003 0.003 0.004 0.002 0.005 0.002 0.004 101

6: 0.005 0.002 0.011 0.007 0.005 0.006 0.005 0.002 0.004 0.003 0.008 0.885 0.030 0.006 0.004 0.002 0.016 20

7: 0.002 0.003 0.003 0.003 0.003 0.003 0.958 0.003 0.004 0.003 0.003 0.003 0.003 0.001 0.002 0.002 0.002 47

8: 0.006 0.005 0.005 0.006 0.007 0.010 0.005 0.004 0.005 0.005 0.007 0.011 0.902 0.002 0.009 0.003 0.011 47

9: 0.004 0.006 0.006 0.004 0.004 0.004 0.003 0.004 0.005 0.006 0.005 0.005 0.007 0.003 0.922 0.004 0.008 45

10: 0.002 0.903 0.003 0.003 0.002 0.002 0.003 0.041 0.016 0.004 0.003 0.002 0.003 0.001 0.003 0.006 0.002 47

11: 0.018 0.003 0.805 0.080 0.017 0.018 0.013 0.004 0.005 0.005 0.004 0.004 0.003 0.002 0.007 0.005 0.006 22

12: 0.002 0.002 0.001 0.001 0.001 0.002 0.002 0.871 0.004 0.002 0.002 0.002 0.001 0.002 0.002 0.102 0.001 26

13: 0.001 0.002 0.001 0.001 0.001 0.002 0.001 0.003 0.001 0.002 0.001 0.001 0.001 0.906 0.002 0.072 0.001 21

14: 0.010 0.005 0.082 0.825 0.010 0.008 0.010 0.010 0.005 0.005 0.004 0.006 0.004 0.002 0.009 0.003 0.004 46

15: 0.009 0.003 0.803 0.071 0.038 0.005 0.041 0.004 0.003 0.003 0.007 0.003 0.002 0.002 0.002 0.002 0.002 23

16: 0.015 0.002 0.006 0.008 0.918 0.008 0.012 0.003 0.004 0.004 0.003 0.003 0.004 0.002 0.003 0.003 0.003 45

17: 0.002 0.002 0.002 0.002 0.002 0.003 0.002 0.002 0.002 0.002 0.963 0.006 0.003 0.001 0.002 0.001 0.003 23

18: 0.019 0.005 0.011 0.034 0.009 0.863 0.007 0.004 0.005 0.005 0.010 0.007 0.005 0.002 0.007 0.003 0.005 45

Run 7:

1: 0.004 0.014 0.005 0.126 0.004 0.010 0.005 0.005 0.002 0.790 0.004 0.004 0.009 0.004 0.005 0.005 0.005 32

2: 0.003 0.003 0.012 0.002 0.944 0.003 0.002 0.002 0.005 0.003 0.002 0.003 0.002 0.004 0.005 0.003 0.002 26

3: 0.004 0.908 0.005 0.011 0.005 0.006 0.006 0.005 0.002 0.010 0.004 0.005 0.005 0.005 0.003 0.014 0.004 45

4: 0.003 0.004 0.007 0.006 0.004 0.005 0.003 0.008 0.002 0.007 0.006 0.860 0.056 0.019 0.002 0.004 0.004 27

5: 0.002 0.003 0.004 0.004 0.004 0.003 0.004 0.004 0.002 0.005 0.003 0.021 0.921 0.008 0.003 0.004 0.005 101

6: 0.002 0.005 0.028 0.007 0.017 0.005 0.005 0.002 0.006 0.011 0.886 0.003 0.004 0.002 0.008 0.006 0.004 20

7: 0.002 0.002 0.003 0.003 0.002 0.003 0.958 0.003 0.001 0.003 0.003 0.003 0.004 0.003 0.003 0.003 0.002 47

8: 0.003 0.006 0.808 0.006 0.097 0.007 0.005 0.006 0.002 0.007 0.011 0.005 0.005 0.004 0.008 0.010 0.009 47

9: 0.004 0.004 0.007 0.004 0.008 0.004 0.003 0.006 0.003 0.006 0.005 0.006 0.005 0.004 0.005 0.004 0.921 45

10: 0.006 0.002 0.003 0.003 0.002 0.002 0.003 0.903 0.002 0.003 0.002 0.004 0.016 0.041 0.003 0.002 0.003 47

11: 0.005 0.017 0.004 0.187 0.006 0.016 0.013 0.003 0.002 0.701 0.004 0.005 0.005 0.004 0.004 0.017 0.006 22

12: 0.102 0.002 0.001 0.001 0.001 0.001 0.002 0.002 0.002 0.001 0.002 0.002 0.004 0.871 0.002 0.002 0.002 26

13: 0.072 0.001 0.001 0.001 0.001 0.001 0.001 0.002 0.905 0.001 0.001 0.002 0.001 0.003 0.001 0.002 0.002 21

14: 0.003 0.011 0.005 0.827 0.005 0.010 0.010 0.005 0.002 0.073 0.006 0.006 0.005 0.010 0.004 0.009 0.009 46

15: 0.002 0.009 0.002 0.175 0.002 0.038 0.042 0.003 0.002 0.699 0.003 0.003 0.003 0.004 0.007 0.005 0.002 23

16: 0.003 0.015 0.004 0.007 0.004 0.918 0.012 0.002 0.002 0.006 0.003 0.004 0.004 0.003 0.003 0.008 0.003 45

17: 0.001 0.002 0.003 0.002 0.003 0.002 0.002 0.002 0.001 0.002 0.006 0.002 0.002 0.002 0.963 0.003 0.002 23

18: 0.003 0.019 0.006 0.033 0.005 0.009 0.007 0.005 0.002 0.011 0.007 0.005 0.005 0.004 0.010 0.863 0.007 45

Run 8:

1: 0.014 0.014 0.003 0.002 0.004 0.005 0.005 0.009 0.010 0.005 0.004 0.005 0.004 0.005 0.004 0.005 0.903 32

2: 0.003 0.002 0.943 0.005 0.003 0.002 0.002 0.002 0.003 0.005 0.003 0.014 0.003 0.003 0.002 0.002 0.003 26

3: 0.909 0.011 0.005 0.002 0.004 0.006 0.004 0.005 0.006 0.003 0.005 0.004 0.005 0.013 0.005 0.005 0.010 45

4: 0.004 0.005 0.004 0.002 0.003 0.003 0.004 0.057 0.006 0.002 0.861 0.006 0.019 0.004 0.006 0.008 0.006 27

5: 0.003 0.004 0.004 0.002 0.002 0.004 0.005 0.922 0.003 0.003 0.021 0.004 0.008 0.004 0.003 0.004 0.005 101

6: 0.005 0.007 0.016 0.006 0.002 0.005 0.004 0.004 0.005 0.008 0.003 0.030 0.002 0.006 0.885 0.002 0.011 20

7: 0.002 0.003 0.002 0.001 0.002 0.958 0.002 0.004 0.003 0.003 0.003 0.003 0.003 0.003 0.003 0.003 0.003 47

8: 0.006 0.006 0.011 0.002 0.003 0.005 0.009 0.005 0.007 0.007 0.005 0.902 0.004 0.010 0.010 0.005 0.005 47

9: 0.004 0.004 0.008 0.003 0.004 0.003 0.922 0.005 0.004 0.005 0.006 0.007 0.004 0.004 0.005 0.006 0.006 45

10: 0.002 0.003 0.002 0.002 0.006 0.003 0.003 0.016 0.002 0.003 0.004 0.003 0.041 0.002 0.002 0.903 0.003 47

11: 0.018 0.083 0.006 0.002 0.005 0.013 0.007 0.005 0.017 0.004 0.005 0.003 0.004 0.018 0.004 0.003 0.802 22

12: 0.002 0.001 0.001 0.002 0.103 0.002 0.002 0.004 0.001 0.002 0.002 0.001 0.871 0.002 0.001 0.002 0.001 26

13: 0.001 0.001 0.001 0.905 0.072 0.001 0.002 0.001 0.001 0.001 0.002 0.001 0.003 0.002 0.001 0.002 0.001 21

14: 0.010 0.826 0.004 0.002 0.003 0.010 0.009 0.005 0.010 0.004 0.005 0.004 0.010 0.008 0.006 0.005 0.081 46

15: 0.009 0.073 0.002 0.002 0.002 0.042 0.002 0.003 0.038 0.007 0.003 0.002 0.004 0.005 0.003 0.003 0.800 23

16: 0.014 0.008 0.003 0.002 0.003 0.012 0.003 0.004 0.918 0.003 0.004 0.004 0.003 0.008 0.003 0.002 0.006 45

17: 0.002 0.002 0.003 0.001 0.001 0.002 0.002 0.002 0.002 0.963 0.002 0.003 0.002 0.003 0.006 0.002 0.002 23

18: 0.019 0.034 0.005 0.002 0.003 0.007 0.007 0.005 0.008 0.010 0.005 0.005 0.004 0.863 0.007 0.005 0.011 45

Run 9:

1: 0.004 0.005 0.006 0.005 0.009 0.004 0.902 0.005 0.014 0.005 0.014 0.004 0.010 0.004 0.002 0.005 0.004 32

2: 0.003 0.005 0.008 0.002 0.002 0.002 0.003 0.002 0.002 0.002 0.003 0.004 0.003 0.002 0.005 0.003 0.947 26

3: 0.004 0.003 0.004 0.004 0.005 0.005 0.010 0.006 0.011 0.005 0.909 0.003 0.006 0.004 0.002 0.013 0.005 45

4: 0.006 0.002 0.012 0.004 0.058 0.858 0.005 0.003 0.005 0.008 0.004 0.009 0.005 0.010 0.002 0.004 0.004 27

5: 0.004 0.003 0.004 0.005 0.918 0.021 0.005 0.004 0.004 0.004 0.003 0.006 0.003 0.007 0.002 0.004 0.004 101

6: 0.002 0.017 0.897 0.005 0.006 0.003 0.011 0.003 0.008 0.002 0.005 0.003 0.018 0.002 0.007 0.006 0.005 20

7: 0.002 0.003 0.003 0.002 0.004 0.003 0.003 0.958 0.003 0.003 0.002 0.004 0.003 0.002 0.001 0.003 0.002 47

8: 0.003 0.007 0.904 0.008 0.004 0.006 0.005 0.006 0.005 0.005 0.008 0.004 0.007 0.004 0.002 0.008 0.015 47

9: 0.004 0.005 0.007 0.922 0.005 0.005 0.006 0.003 0.004 0.006 0.004 0.004 0.004 0.004 0.003 0.004 0.008 45

10: 0.005 0.003 0.002 0.003 0.013 0.004 0.003 0.003 0.003 0.905 0.002 0.041 0.002 0.005 0.002 0.002 0.002 47

11: 0.006 0.004 0.004 0.007 0.004 0.005 0.802 0.013 0.081 0.003 0.018 0.004 0.017 0.004 0.002 0.018 0.006 22

12: 0.145 0.001 0.001 0.002 0.003 0.002 0.001 0.002 0.001 0.002 0.001 0.323 0.001 0.510 0.001 0.001 0.001 26

13: 0.068 0.001 0.001 0.002 0.001 0.002 0.001 0.001 0.001 0.002 0.001 0.002 0.001 0.002 0.909 0.002 0.001 21

14: 0.003 0.005 0.006 0.010 0.005 0.006 0.080 0.010 0.826 0.005 0.010 0.005 0.010 0.007 0.002 0.008 0.004 46

15: 0.002 0.007 0.002 0.002 0.003 0.003 0.801 0.041 0.072 0.003 0.009 0.005 0.038 0.003 0.002 0.005 0.002 23

16: 0.003 0.003 0.004 0.003 0.004 0.004 0.006 0.012 0.008 0.002 0.014 0.003 0.918 0.003 0.002 0.008 0.004 45

17: 0.001 0.964 0.006 0.003 0.002 0.002 0.002 0.002 0.002 0.002 0.002 0.002 0.002 0.002 0.001 0.003 0.003 23

18: 0.003 0.010 0.006 0.007 0.005 0.005 0.010 0.007 0.034 0.005 0.019 0.003 0.009 0.004 0.002 0.864 0.005 45

Run 10:

1: 0.005 0.004 0.005 0.014 0.005 0.005 0.003 0.004 0.010 0.012 0.903 0.005 0.005 0.005 0.009 0.002 0.004 32

2: 0.002 0.002 0.002 0.002 0.002 0.002 0.943 0.002 0.003 0.003 0.003 0.014 0.005 0.003 0.002 0.005 0.004 26

3: 0.005 0.005 0.004 0.011 0.004 0.006 0.005 0.004 0.006 0.908 0.010 0.004 0.003 0.013 0.005 0.002 0.005 45

4: 0.009 0.476 0.364 0.006 0.004 0.002 0.003 0.008 0.005 0.006 0.006 0.008 0.002 0.005 0.071 0.002 0.023 27

5: 0.004 0.016 0.014 0.004 0.005 0.004 0.004 0.003 0.003 0.004 0.005 0.004 0.003 0.004 0.916 0.002 0.007 101

6: 0.002 0.002 0.003 0.007 0.004 0.005 0.016 0.884 0.005 0.005 0.011 0.030 0.008 0.006 0.004 0.006 0.002 20

7: 0.003 0.003 0.003 0.003 0.002 0.958 0.002 0.003 0.003 0.002 0.003 0.003 0.003 0.003 0.004 0.001 0.003 47

8: 0.005 0.005 0.005 0.006 0.008 0.005 0.011 0.011 0.007 0.006 0.005 0.900 0.007 0.010 0.004 0.002 0.004 47

9: 0.006 0.005 0.005 0.004 0.920 0.003 0.008 0.005 0.004 0.004 0.006 0.008 0.005 0.004 0.005 0.003 0.005 45

10: 0.906 0.004 0.003 0.003 0.003 0.003 0.002 0.002 0.002 0.002 0.003 0.003 0.003 0.003 0.017 0.001 0.038 47

11: 0.003 0.005 0.006 0.078 0.007 0.013 0.006 0.004 0.017 0.018 0.806 0.003 0.004 0.018 0.004 0.002 0.005 22

12: 0.003 0.008 0.005 0.002 0.003 0.002 0.001 0.002 0.002 0.002 0.002 0.002 0.002 0.002 0.005 0.006 0.952 26

13: 0.003 0.002 0.002 0.001 0.002 0.002 0.001 0.003 0.002 0.002 0.001 0.002 0.001 0.003 0.002 0.963 0.009 21

14: 0.005 0.006 0.004 0.824 0.009 0.010 0.004 0.006 0.010 0.010 0.081 0.004 0.005 0.008 0.005 0.002 0.009 46

15: 0.003 0.004 0.002 0.072 0.002 0.042 0.002 0.003 0.037 0.010 0.801 0.002 0.007 0.005 0.003 0.002 0.004 23

16: 0.002 0.005 0.004 0.008 0.003 0.012 0.003 0.003 0.916 0.015 0.006 0.004 0.003 0.008 0.004 0.002 0.003 45

17: 0.002 0.002 0.003 0.002 0.002 0.002 0.003 0.006 0.002 0.002 0.002 0.003 0.962 0.003 0.002 0.001 0.002 23

18: 0.005 0.004 0.005 0.034 0.007 0.007 0.005 0.007 0.009 0.019 0.011 0.005 0.010 0.862 0.005 0.003 0.003 45
