# Supplementary material for: Genetic Diversity and Population Structure of Dülmen Wild, Liebenthal and Polish Konik Horses in Comparison with Przewalski, Sorraia, German Draught and Riding Horses
Source: Animals (Basel). 2024 Jul 31;14(15):2221. doi: 10.3390/ani14152221 (PMC11311111; doi:10.3390/ani14152221)
Supplement: Supplementary file 1 [file animals-14-02221-s001.zip › Supplementary-Table-S10-K18-membership-coefficients.docx]

**Table S10.** Membership coefficients from 10 independent STRUCTURE runs for 18 horse populations for K = 18.

| **Population** | **Abbreviation** | **Running number** |
| --- | --- | --- |
| Altmaerkisch Coldblood | AL | 1 |
| Arabian | AR | 2 |
| Black Forest Horse | BF | 3 |
| Dülmen Horse | DH | 4 |
| Dülmen Wild Horse | DW | 5 |
| Exmoor Pony | EX | 6 |
| Friesian | FR | 7 |
| Hanoverian Warmblood | HA | 8 |
| Icelandic Horse | IC | 9 |
| Liebenthal Horse | LH | 10 |
| Mecklenburg Coldblood | ME | 11 |
| Polish Konik Horse | KO | 12 |
| Przewalski Horse | PR | 13 |
| Rhenish German Coldblood | RG | 14 |
| Saxon-Thuringa Coldblood | ST | 15 |
| Schleswig Draught Horse | SL | 16 |
| Sorraia Horse | SO | 17 |
| South German Coldblood | SG | 18 |

Run 1: Given Inferred Clusters Number of

Pop 1 2 3 4 5 6 7 8 9 10 11 12 13 14 15 16 17 18 Individuals

1: 0.004 0.003 0.009 0.014 0.004 0.004 0.900 0.005 0.014 0.002 0.004 0.005 0.010 0.005 0.005 0.005 0.005 0.004 32

2: 0.002 0.940 0.002 0.002 0.003 0.005 0.003 0.002 0.003 0.005 0.002 0.002 0.003 0.002 0.005 0.014 0.003 0.002 26

3: 0.005 0.005 0.005 0.011 0.004 0.003 0.010 0.004 0.906 0.002 0.004 0.004 0.006 0.006 0.003 0.004 0.013 0.004 45

4: 0.858 0.004 0.057 0.005 0.006 0.009 0.005 0.008 0.004 0.002 0.006 0.004 0.005 0.003 0.002 0.006 0.004 0.011 27

5: 0.021 0.004 0.916 0.004 0.004 0.006 0.005 0.004 0.003 0.002 0.003 0.005 0.003 0.004 0.003 0.004 0.004 0.007 101

6: 0.003 0.016 0.004 0.006 0.002 0.002 0.011 0.002 0.005 0.006 0.884 0.004 0.005 0.005 0.008 0.030 0.006 0.002 20

7: 0.003 0.002 0.004 0.003 0.002 0.004 0.003 0.003 0.002 0.001 0.003 0.002 0.003 0.956 0.003 0.003 0.003 0.002 47

8: 0.005 0.011 0.004 0.006 0.003 0.004 0.005 0.005 0.006 0.002 0.010 0.009 0.007 0.005 0.007 0.898 0.010 0.004 47

9: 0.006 0.008 0.005 0.004 0.004 0.004 0.006 0.006 0.004 0.003 0.005 0.917 0.004 0.003 0.005 0.007 0.004 0.004 45

10: 0.004 0.002 0.013 0.003 0.005 0.039 0.003 0.905 0.002 0.001 0.002 0.003 0.002 0.003 0.003 0.003 0.002 0.005 47

11: 0.005 0.006 0.005 0.082 0.006 0.004 0.799 0.003 0.018 0.002 0.004 0.007 0.017 0.013 0.004 0.003 0.018 0.004 22

12: 0.002 0.001 0.003 0.001 0.144 0.336 0.001 0.002 0.001 0.001 0.001 0.002 0.001 0.002 0.001 0.001 0.001 0.495 26

13: 0.002 0.001 0.001 0.001 0.068 0.002 0.001 0.002 0.001 0.908 0.001 0.002 0.001 0.001 0.001 0.001 0.002 0.002 21

14: 0.005 0.004 0.005 0.824 0.003 0.004 0.080 0.005 0.010 0.002 0.006 0.010 0.010 0.009 0.005 0.004 0.008 0.007 46

15: 0.003 0.002 0.003 0.072 0.002 0.005 0.799 0.003 0.010 0.002 0.003 0.002 0.038 0.041 0.007 0.002 0.005 0.003 23

16: 0.004 0.003 0.004 0.008 0.003 0.003 0.006 0.002 0.015 0.002 0.003 0.003 0.915 0.012 0.003 0.004 0.008 0.003 45

17: 0.002 0.003 0.002 0.002 0.001 0.002 0.002 0.002 0.002 0.001 0.006 0.002 0.002 0.002 0.961 0.003 0.003 0.002 23

18: 0.005 0.005 0.005 0.034 0.003 0.003 0.011 0.005 0.019 0.002 0.007 0.007 0.009 0.007 0.010 0.005 0.860 0.004 45

Run 2

1: 0.011 0.004 0.004 0.004 0.004 0.013 0.004 0.003 0.007 0.005 0.002 0.002 0.004 0.003 0.759 0.010 0.155 0.005 32

2: 0.003 0.003 0.002 0.002 0.003 0.003 0.941 0.002 0.002 0.002 0.005 0.003 0.014 0.005 0.003 0.002 0.002 0.003 26

3: 0.903 0.005 0.006 0.004 0.005 0.006 0.005 0.005 0.005 0.005 0.002 0.004 0.004 0.003 0.009 0.010 0.008 0.013 45

4: 0.004 0.019 0.003 0.004 0.859 0.005 0.004 0.008 0.056 0.006 0.002 0.003 0.006 0.002 0.006 0.005 0.005 0.004 27

5: 0.003 0.008 0.004 0.005 0.021 0.003 0.004 0.004 0.918 0.003 0.002 0.002 0.004 0.003 0.005 0.004 0.003 0.004 101

6: 0.004 0.002 0.005 0.004 0.003 0.005 0.016 0.002 0.004 0.882 0.006 0.002 0.030 0.008 0.007 0.006 0.009 0.006 20

7: 0.002 0.003 0.955 0.002 0.003 0.003 0.002 0.003 0.004 0.003 0.001 0.002 0.003 0.003 0.003 0.003 0.002 0.003 47

8: 0.006 0.004 0.005 0.009 0.005 0.007 0.011 0.005 0.005 0.010 0.002 0.003 0.898 0.007 0.004 0.006 0.004 0.009 47

9: 0.004 0.004 0.003 0.917 0.006 0.004 0.008 0.006 0.005 0.005 0.003 0.004 0.007 0.005 0.007 0.004 0.004 0.004 45

10: 0.002 0.040 0.003 0.003 0.004 0.002 0.002 0.901 0.016 0.002 0.002 0.006 0.003 0.003 0.003 0.003 0.003 0.002 47

11: 0.025 0.004 0.014 0.007 0.005 0.011 0.005 0.003 0.005 0.004 0.002 0.006 0.004 0.004 0.072 0.069 0.745 0.016 22

12: 0.002 0.869 0.002 0.002 0.002 0.001 0.001 0.002 0.005 0.002 0.002 0.103 0.001 0.002 0.001 0.001 0.001 0.002 26

13: 0.001 0.003 0.001 0.002 0.002 0.001 0.001 0.002 0.001 0.001 0.905 0.071 0.001 0.001 0.001 0.001 0.001 0.002 21

14: 0.009 0.009 0.010 0.009 0.006 0.010 0.004 0.005 0.005 0.006 0.002 0.003 0.004 0.005 0.059 0.816 0.031 0.008 46

15: 0.011 0.004 0.046 0.002 0.003 0.042 0.002 0.003 0.003 0.002 0.002 0.002 0.003 0.006 0.099 0.070 0.695 0.005 23

16: 0.015 0.003 0.012 0.003 0.004 0.914 0.004 0.002 0.004 0.003 0.002 0.003 0.004 0.003 0.005 0.007 0.006 0.008 45

17: 0.002 0.002 0.002 0.002 0.002 0.002 0.003 0.002 0.002 0.006 0.001 0.001 0.003 0.961 0.002 0.002 0.002 0.003 23

18: 0.019 0.004 0.007 0.007 0.005 0.009 0.005 0.005 0.005 0.007 0.002 0.003 0.005 0.010 0.010 0.033 0.008 0.858 45

Run 3

1: 0.004 0.004 0.009 0.004 0.004 0.005 0.002 0.010 0.013 0.005 0.004 0.900 0.005 0.005 0.005 0.003 0.005 0.014 32

2: 0.005 0.002 0.002 0.002 0.003 0.014 0.005 0.003 0.003 0.002 0.002 0.003 0.002 0.005 0.002 0.940 0.003 0.002 26

3: 0.003 0.004 0.005 0.005 0.004 0.004 0.002 0.006 0.906 0.006 0.004 0.010 0.004 0.003 0.004 0.005 0.013 0.011 45

4: 0.009 0.011 0.057 0.858 0.006 0.006 0.002 0.005 0.004 0.003 0.006 0.005 0.004 0.002 0.008 0.004 0.004 0.005 27

5: 0.006 0.007 0.916 0.021 0.004 0.004 0.002 0.003 0.003 0.004 0.003 0.005 0.005 0.003 0.004 0.004 0.004 0.004 101

6: 0.002 0.002 0.004 0.003 0.002 0.030 0.006 0.005 0.005 0.005 0.884 0.010 0.004 0.008 0.002 0.016 0.006 0.007 20

7: 0.004 0.002 0.004 0.003 0.002 0.003 0.001 0.003 0.002 0.956 0.003 0.003 0.002 0.003 0.003 0.002 0.003 0.003 47

8: 0.004 0.004 0.004 0.005 0.003 0.898 0.002 0.007 0.006 0.005 0.010 0.005 0.009 0.007 0.005 0.011 0.010 0.006 47

9: 0.004 0.004 0.005 0.006 0.004 0.007 0.003 0.004 0.004 0.003 0.005 0.006 0.917 0.005 0.006 0.008 0.004 0.004 45

10: 0.039 0.005 0.013 0.004 0.005 0.003 0.002 0.002 0.002 0.003 0.002 0.003 0.003 0.003 0.905 0.002 0.002 0.002 47

11: 0.004 0.004 0.004 0.005 0.006 0.003 0.002 0.018 0.018 0.013 0.004 0.798 0.007 0.004 0.003 0.006 0.018 0.082 22

12: 0.337 0.495 0.003 0.002 0.144 0.001 0.001 0.001 0.001 0.002 0.001 0.001 0.002 0.001 0.002 0.001 0.001 0.001 26

13: 0.002 0.002 0.001 0.002 0.067 0.001 0.908 0.001 0.001 0.001 0.001 0.001 0.002 0.001 0.002 0.001 0.002 0.001 21

14: 0.004 0.007 0.005 0.005 0.003 0.004 0.002 0.010 0.010 0.010 0.006 0.080 0.010 0.005 0.005 0.004 0.008 0.823 46

15: 0.005 0.003 0.003 0.003 0.002 0.002 0.002 0.039 0.009 0.041 0.003 0.799 0.002 0.007 0.003 0.002 0.005 0.071 23

16: 0.003 0.003 0.004 0.005 0.003 0.004 0.002 0.916 0.015 0.012 0.003 0.006 0.003 0.003 0.002 0.003 0.008 0.008 45

17: 0.002 0.002 0.002 0.002 0.001 0.003 0.001 0.002 0.002 0.002 0.006 0.002 0.002 0.961 0.002 0.003 0.003 0.002 23

18: 0.003 0.004 0.005 0.005 0.003 0.005 0.002 0.009 0.019 0.007 0.007 0.011 0.007 0.010 0.005 0.005 0.860 0.034 45

Run 4:

1: 0.009 0.004 0.005 0.005 0.005 0.004 0.004 0.010 0.014 0.002 0.004 0.003 0.004 0.900 0.005 0.014 0.005 0.005 32

2: 0.002 0.005 0.003 0.002 0.002 0.003 0.002 0.003 0.002 0.005 0.002 0.940 0.002 0.003 0.014 0.003 0.005 0.002 26

3: 0.005 0.003 0.013 0.006 0.005 0.004 0.005 0.006 0.011 0.002 0.005 0.005 0.004 0.010 0.004 0.906 0.003 0.004 45

4: 0.056 0.009 0.004 0.003 0.008 0.006 0.859 0.005 0.005 0.002 0.006 0.004 0.011 0.005 0.006 0.004 0.002 0.004 27

5: 0.916 0.006 0.004 0.004 0.004 0.004 0.021 0.003 0.004 0.002 0.003 0.004 0.007 0.005 0.004 0.003 0.003 0.005 101

6: 0.004 0.002 0.006 0.005 0.002 0.002 0.003 0.005 0.007 0.006 0.884 0.016 0.002 0.010 0.030 0.005 0.008 0.004 20

7: 0.004 0.004 0.003 0.956 0.003 0.002 0.003 0.003 0.003 0.001 0.003 0.002 0.002 0.003 0.003 0.002 0.003 0.002 47

8: 0.005 0.004 0.010 0.005 0.005 0.003 0.005 0.007 0.006 0.002 0.011 0.011 0.004 0.005 0.898 0.006 0.007 0.009 47

9: 0.005 0.004 0.004 0.003 0.006 0.004 0.006 0.004 0.004 0.003 0.005 0.008 0.004 0.006 0.007 0.004 0.005 0.917 45

10: 0.014 0.039 0.002 0.003 0.905 0.005 0.004 0.002 0.003 0.002 0.002 0.002 0.005 0.003 0.003 0.002 0.003 0.003 47

11: 0.005 0.004 0.018 0.013 0.003 0.006 0.005 0.017 0.084 0.002 0.004 0.006 0.004 0.798 0.003 0.018 0.004 0.007 22

12: 0.003 0.338 0.001 0.002 0.002 0.144 0.002 0.001 0.001 0.001 0.001 0.001 0.495 0.001 0.001 0.001 0.001 0.002 26

13: 0.001 0.002 0.002 0.001 0.002 0.068 0.002 0.001 0.001 0.908 0.001 0.001 0.002 0.001 0.001 0.001 0.001 0.002 21

14: 0.005 0.004 0.008 0.010 0.005 0.003 0.005 0.010 0.824 0.002 0.006 0.004 0.007 0.080 0.004 0.010 0.005 0.009 46

15: 0.003 0.005 0.005 0.042 0.003 0.002 0.003 0.038 0.072 0.002 0.003 0.002 0.003 0.798 0.002 0.010 0.007 0.002 23

16: 0.004 0.003 0.008 0.012 0.002 0.003 0.004 0.915 0.008 0.002 0.003 0.003 0.003 0.006 0.004 0.015 0.003 0.003 45

17: 0.002 0.002 0.003 0.002 0.002 0.001 0.002 0.002 0.002 0.001 0.006 0.003 0.002 0.002 0.003 0.002 0.961 0.002 23

18: 0.005 0.003 0.860 0.007 0.005 0.003 0.005 0.008 0.034 0.002 0.007 0.005 0.004 0.011 0.005 0.019 0.010 0.007 45

Run 5:

1: 0.013 0.005 0.005 0.004 0.013 0.005 0.005 0.004 0.010 0.002 0.003 0.004 0.005 0.013 0.009 0.004 0.005 0.891 32

2: 0.003 0.002 0.002 0.003 0.003 0.014 0.003 0.002 0.003 0.005 0.940 0.003 0.005 0.002 0.002 0.003 0.002 0.003 26

3: 0.019 0.004 0.005 0.005 0.896 0.004 0.012 0.005 0.006 0.002 0.005 0.004 0.003 0.007 0.005 0.005 0.006 0.009 45

4: 0.007 0.004 0.008 0.019 0.004 0.006 0.004 0.006 0.005 0.002 0.004 0.003 0.002 0.005 0.055 0.859 0.003 0.005 27

5: 0.005 0.005 0.004 0.008 0.003 0.004 0.004 0.003 0.003 0.002 0.004 0.002 0.003 0.003 0.917 0.021 0.004 0.005 101

6: 0.003 0.004 0.002 0.002 0.004 0.030 0.006 0.882 0.005 0.006 0.016 0.002 0.008 0.007 0.004 0.003 0.005 0.011 20

7: 0.003 0.002 0.003 0.003 0.002 0.003 0.003 0.003 0.003 0.001 0.002 0.002 0.003 0.003 0.004 0.003 0.955 0.003 47

8: 0.005 0.009 0.005 0.004 0.006 0.897 0.009 0.010 0.007 0.002 0.011 0.003 0.007 0.006 0.004 0.005 0.005 0.005 47

9: 0.003 0.918 0.006 0.004 0.004 0.007 0.004 0.005 0.004 0.003 0.008 0.004 0.005 0.005 0.005 0.006 0.003 0.006 45

10: 0.003 0.003 0.901 0.041 0.002 0.003 0.002 0.002 0.002 0.001 0.002 0.006 0.003 0.002 0.015 0.004 0.003 0.003 47

11: 0.019 0.005 0.003 0.004 0.017 0.003 0.019 0.004 0.017 0.002 0.006 0.005 0.004 0.063 0.004 0.005 0.013 0.807 22

12: 0.002 0.002 0.002 0.869 0.002 0.001 0.002 0.002 0.001 0.002 0.001 0.102 0.002 0.001 0.005 0.002 0.002 0.001 26

13: 0.002 0.002 0.002 0.003 0.001 0.001 0.002 0.001 0.001 0.903 0.001 0.071 0.001 0.001 0.001 0.002 0.001 0.001 21

14: 0.150 0.009 0.005 0.010 0.008 0.003 0.007 0.006 0.009 0.002 0.004 0.003 0.005 0.669 0.005 0.006 0.008 0.092 46

15: 0.029 0.002 0.003 0.004 0.009 0.002 0.006 0.003 0.038 0.002 0.002 0.002 0.007 0.055 0.003 0.003 0.041 0.790 23

16: 0.007 0.003 0.002 0.003 0.015 0.004 0.008 0.003 0.911 0.002 0.003 0.003 0.003 0.007 0.004 0.004 0.012 0.006 45

17: 0.002 0.002 0.002 0.002 0.002 0.003 0.003 0.006 0.002 0.001 0.003 0.001 0.961 0.002 0.002 0.002 0.002 0.002 23

18: 0.008 0.007 0.005 0.004 0.020 0.005 0.858 0.007 0.008 0.002 0.005 0.003 0.010 0.029 0.005 0.005 0.007 0.012 45

Run 6:

1: 0.004 0.013 0.002 0.004 0.013 0.009 0.005 0.892 0.004 0.005 0.005 0.003 0.013 0.005 0.005 0.010 0.005 0.004 32

2: 0.003 0.003 0.005 0.003 0.003 0.002 0.002 0.003 0.002 0.014 0.002 0.940 0.002 0.005 0.002 0.003 0.003 0.003 26

3: 0.004 0.896 0.002 0.005 0.018 0.005 0.006 0.009 0.004 0.004 0.004 0.005 0.007 0.003 0.005 0.006 0.012 0.005 45

4: 0.003 0.004 0.002 0.859 0.007 0.055 0.003 0.005 0.006 0.006 0.004 0.004 0.004 0.002 0.008 0.005 0.004 0.019 27

5: 0.002 0.003 0.002 0.021 0.005 0.917 0.004 0.005 0.003 0.004 0.005 0.004 0.003 0.003 0.004 0.003 0.003 0.008 101

6: 0.002 0.004 0.006 0.003 0.003 0.004 0.005 0.011 0.881 0.030 0.004 0.017 0.007 0.008 0.002 0.005 0.006 0.002 20

7: 0.002 0.002 0.001 0.003 0.003 0.004 0.955 0.003 0.003 0.003 0.002 0.002 0.003 0.003 0.003 0.003 0.003 0.003 47

8: 0.003 0.006 0.002 0.005 0.005 0.005 0.005 0.005 0.010 0.897 0.009 0.011 0.006 0.007 0.005 0.007 0.009 0.004 47

9: 0.004 0.004 0.003 0.006 0.003 0.005 0.003 0.006 0.005 0.007 0.918 0.008 0.005 0.005 0.006 0.004 0.004 0.004 45

10: 0.006 0.002 0.001 0.004 0.003 0.016 0.003 0.003 0.002 0.003 0.003 0.002 0.002 0.003 0.900 0.002 0.002 0.041 47

11: 0.005 0.017 0.002 0.005 0.018 0.005 0.013 0.809 0.004 0.003 0.006 0.006 0.061 0.004 0.003 0.017 0.018 0.004 22

12: 0.102 0.002 0.002 0.002 0.002 0.005 0.002 0.001 0.002 0.001 0.002 0.001 0.001 0.002 0.002 0.001 0.002 0.870 26

13: 0.072 0.001 0.903 0.002 0.002 0.001 0.001 0.001 0.001 0.001 0.002 0.001 0.001 0.001 0.002 0.001 0.002 0.002 21

14: 0.003 0.008 0.002 0.006 0.149 0.005 0.009 0.092 0.006 0.003 0.009 0.004 0.668 0.005 0.005 0.009 0.007 0.010 46

15: 0.002 0.009 0.002 0.003 0.029 0.003 0.041 0.791 0.003 0.002 0.002 0.002 0.053 0.007 0.003 0.038 0.006 0.004 23

16: 0.003 0.015 0.002 0.004 0.007 0.004 0.012 0.006 0.003 0.004 0.003 0.004 0.007 0.003 0.002 0.911 0.008 0.003 45

17: 0.001 0.002 0.001 0.002 0.002 0.002 0.002 0.002 0.006 0.003 0.002 0.003 0.002 0.961 0.002 0.002 0.003 0.002 23

18: 0.003 0.020 0.002 0.005 0.008 0.005 0.007 0.012 0.007 0.005 0.007 0.005 0.029 0.010 0.005 0.008 0.858 0.004 45

Run 7:

1: 0.004 0.004 0.002 0.005 0.004 0.009 0.900 0.005 0.003 0.004 0.005 0.005 0.014 0.010 0.005 0.005 0.014 0.004 32

2: 0.002 0.002 0.005 0.005 0.003 0.002 0.003 0.002 0.940 0.005 0.002 0.002 0.002 0.003 0.003 0.014 0.003 0.002 26

3: 0.004 0.004 0.002 0.003 0.004 0.005 0.010 0.004 0.005 0.003 0.006 0.005 0.011 0.006 0.013 0.004 0.906 0.005 45

4: 0.011 0.006 0.002 0.002 0.006 0.057 0.005 0.004 0.004 0.009 0.003 0.008 0.005 0.005 0.004 0.006 0.004 0.858 27

5: 0.007 0.003 0.002 0.003 0.004 0.916 0.005 0.005 0.004 0.006 0.004 0.004 0.004 0.003 0.004 0.004 0.003 0.021 101

6: 0.002 0.884 0.006 0.008 0.002 0.004 0.010 0.004 0.016 0.002 0.005 0.002 0.007 0.005 0.006 0.030 0.004 0.003 20

7: 0.002 0.003 0.001 0.003 0.002 0.004 0.003 0.002 0.002 0.004 0.956 0.003 0.003 0.003 0.003 0.003 0.002 0.003 47

8: 0.004 0.010 0.002 0.007 0.003 0.004 0.005 0.009 0.011 0.004 0.005 0.005 0.006 0.007 0.010 0.898 0.006 0.005 47

9: 0.004 0.005 0.003 0.005 0.004 0.005 0.006 0.917 0.008 0.004 0.003 0.006 0.004 0.004 0.004 0.007 0.004 0.005 45

10: 0.005 0.002 0.001 0.003 0.005 0.013 0.003 0.003 0.002 0.039 0.003 0.905 0.003 0.002 0.002 0.003 0.002 0.004 47

11: 0.004 0.004 0.002 0.004 0.006 0.004 0.799 0.007 0.006 0.004 0.013 0.003 0.082 0.017 0.018 0.003 0.018 0.005 22

12: 0.495 0.001 0.001 0.001 0.145 0.003 0.001 0.002 0.001 0.336 0.002 0.002 0.001 0.001 0.001 0.001 0.001 0.002 26

13: 0.002 0.001 0.908 0.001 0.067 0.001 0.001 0.002 0.001 0.002 0.001 0.002 0.001 0.001 0.002 0.001 0.001 0.002 21

14: 0.007 0.006 0.002 0.004 0.003 0.005 0.080 0.010 0.004 0.004 0.010 0.005 0.824 0.010 0.008 0.004 0.010 0.005 46

15: 0.003 0.003 0.002 0.007 0.002 0.003 0.797 0.002 0.002 0.005 0.041 0.003 0.073 0.038 0.005 0.002 0.010 0.003 23

16: 0.003 0.003 0.002 0.003 0.003 0.004 0.006 0.003 0.003 0.003 0.012 0.002 0.008 0.915 0.008 0.004 0.015 0.004 45

17: 0.002 0.006 0.001 0.961 0.001 0.002 0.002 0.002 0.003 0.002 0.002 0.002 0.002 0.002 0.003 0.003 0.002 0.002 23

18: 0.004 0.007 0.002 0.010 0.003 0.005 0.010 0.007 0.005 0.003 0.007 0.005 0.034 0.009 0.860 0.005 0.019 0.005 45

Run 8:

1: 0.002 0.900 0.004 0.005 0.004 0.004 0.009 0.005 0.005 0.014 0.003 0.014 0.010 0.005 0.004 0.005 0.004 0.005 32

2: 0.005 0.003 0.003 0.005 0.002 0.005 0.002 0.002 0.002 0.002 0.940 0.003 0.003 0.003 0.002 0.014 0.002 0.002 26

3: 0.002 0.010 0.004 0.003 0.004 0.003 0.005 0.006 0.004 0.011 0.005 0.906 0.006 0.013 0.005 0.004 0.004 0.004 45

4: 0.002 0.005 0.006 0.002 0.006 0.009 0.057 0.003 0.004 0.005 0.004 0.004 0.005 0.004 0.858 0.006 0.011 0.008 27

5: 0.002 0.005 0.004 0.003 0.003 0.006 0.916 0.004 0.005 0.004 0.004 0.003 0.003 0.004 0.021 0.004 0.007 0.004 101

6: 0.006 0.010 0.002 0.008 0.884 0.002 0.004 0.005 0.004 0.007 0.016 0.005 0.005 0.006 0.003 0.030 0.002 0.002 20

7: 0.001 0.003 0.002 0.003 0.003 0.004 0.004 0.956 0.002 0.003 0.002 0.002 0.003 0.003 0.003 0.003 0.002 0.003 47

8: 0.002 0.005 0.003 0.007 0.010 0.004 0.004 0.005 0.009 0.006 0.011 0.006 0.007 0.010 0.005 0.898 0.004 0.005 47

9: 0.003 0.006 0.004 0.005 0.005 0.004 0.005 0.003 0.917 0.004 0.008 0.004 0.004 0.004 0.006 0.007 0.004 0.006 45

10: 0.002 0.003 0.005 0.003 0.002 0.039 0.013 0.003 0.003 0.003 0.002 0.002 0.002 0.002 0.004 0.003 0.005 0.905 47

11: 0.002 0.798 0.006 0.004 0.004 0.004 0.004 0.013 0.006 0.083 0.006 0.018 0.017 0.018 0.005 0.003 0.004 0.003 22

12: 0.001 0.001 0.144 0.001 0.001 0.337 0.003 0.002 0.002 0.001 0.001 0.001 0.001 0.001 0.002 0.001 0.495 0.002 26

13: 0.908 0.001 0.067 0.001 0.001 0.002 0.001 0.001 0.002 0.001 0.001 0.001 0.001 0.002 0.002 0.001 0.002 0.002 21

14: 0.002 0.083 0.003 0.004 0.006 0.005 0.005 0.010 0.010 0.820 0.004 0.010 0.010 0.008 0.005 0.004 0.007 0.005 46

15: 0.002 0.798 0.002 0.007 0.003 0.005 0.003 0.041 0.002 0.072 0.002 0.010 0.037 0.005 0.003 0.002 0.003 0.003 23

16: 0.002 0.006 0.003 0.003 0.003 0.003 0.004 0.012 0.003 0.008 0.004 0.014 0.916 0.008 0.004 0.004 0.003 0.002 45

17: 0.001 0.002 0.001 0.961 0.006 0.002 0.002 0.002 0.002 0.002 0.003 0.002 0.002 0.003 0.002 0.003 0.002 0.002 23

18: 0.002 0.011 0.003 0.010 0.007 0.003 0.005 0.007 0.007 0.034 0.005 0.019 0.009 0.860 0.005 0.005 0.004 0.005 45

Run 9:

1: 0.005 0.900 0.014 0.004 0.005 0.010 0.003 0.002 0.014 0.005 0.005 0.004 0.004 0.009 0.005 0.004 0.005 0.004 32

2: 0.003 0.003 0.002 0.004 0.002 0.003 0.941 0.005 0.003 0.002 0.005 0.003 0.002 0.002 0.014 0.003 0.002 0.002 26

3: 0.013 0.010 0.011 0.004 0.005 0.006 0.005 0.002 0.906 0.006 0.003 0.005 0.005 0.005 0.004 0.004 0.004 0.005 45

4: 0.004 0.005 0.005 0.011 0.007 0.005 0.004 0.002 0.004 0.003 0.002 0.013 0.006 0.057 0.006 0.005 0.004 0.856 27

5: 0.004 0.005 0.004 0.006 0.004 0.003 0.004 0.002 0.003 0.004 0.003 0.007 0.003 0.917 0.004 0.003 0.005 0.021 101

6: 0.006 0.011 0.007 0.002 0.002 0.005 0.016 0.006 0.005 0.005 0.008 0.002 0.883 0.004 0.030 0.002 0.004 0.003 20

7: 0.003 0.003 0.003 0.004 0.003 0.003 0.002 0.001 0.002 0.956 0.003 0.003 0.003 0.004 0.003 0.002 0.002 0.003 47

8: 0.010 0.005 0.006 0.005 0.005 0.007 0.011 0.002 0.006 0.005 0.007 0.004 0.011 0.004 0.897 0.003 0.009 0.005 47

9: 0.004 0.006 0.004 0.005 0.006 0.004 0.008 0.003 0.004 0.003 0.005 0.004 0.005 0.005 0.007 0.004 0.917 0.006 45

10: 0.002 0.003 0.003 0.068 0.866 0.002 0.002 0.002 0.002 0.003 0.003 0.017 0.002 0.012 0.002 0.005 0.003 0.004 47

11: 0.018 0.800 0.081 0.004 0.003 0.017 0.006 0.002 0.018 0.013 0.004 0.004 0.004 0.004 0.003 0.005 0.007 0.005 22

12: 0.001 0.001 0.001 0.216 0.002 0.001 0.001 0.001 0.001 0.002 0.002 0.629 0.001 0.004 0.001 0.130 0.002 0.002 26

13: 0.002 0.001 0.001 0.002 0.002 0.001 0.001 0.906 0.001 0.001 0.001 0.002 0.001 0.001 0.001 0.069 0.002 0.002 21

14: 0.008 0.081 0.822 0.004 0.005 0.010 0.004 0.002 0.010 0.010 0.004 0.008 0.006 0.005 0.004 0.003 0.010 0.005 46

15: 0.005 0.800 0.071 0.004 0.003 0.038 0.002 0.002 0.009 0.041 0.007 0.003 0.003 0.003 0.002 0.002 0.002 0.003 23

16: 0.008 0.006 0.007 0.003 0.002 0.916 0.003 0.002 0.014 0.012 0.003 0.003 0.003 0.004 0.004 0.003 0.003 0.004 45

17: 0.003 0.002 0.002 0.002 0.002 0.002 0.003 0.001 0.002 0.002 0.961 0.002 0.006 0.002 0.003 0.001 0.002 0.002 23

18: 0.859 0.011 0.034 0.004 0.005 0.009 0.005 0.002 0.019 0.007 0.010 0.004 0.007 0.005 0.005 0.003 0.007 0.005 45

Run 10:

1: 0.005 0.014 0.003 0.005 0.005 0.005 0.004 0.009 0.004 0.005 0.014 0.002 0.004 0.005 0.004 0.010 0.004 0.900 32

2: 0.014 0.003 0.940 0.005 0.002 0.002 0.003 0.002 0.002 0.003 0.002 0.005 0.002 0.002 0.004 0.003 0.002 0.003 26

3: 0.004 0.906 0.005 0.003 0.006 0.004 0.004 0.005 0.004 0.013 0.011 0.002 0.005 0.004 0.004 0.006 0.004 0.010 45

4: 0.006 0.004 0.004 0.002 0.003 0.004 0.010 0.056 0.006 0.004 0.005 0.002 0.857 0.008 0.010 0.005 0.010 0.005 27

5: 0.004 0.003 0.004 0.003 0.004 0.005 0.004 0.916 0.003 0.004 0.004 0.002 0.021 0.004 0.005 0.003 0.007 0.005 101

6: 0.030 0.005 0.016 0.008 0.005 0.004 0.002 0.004 0.883 0.006 0.007 0.006 0.003 0.002 0.002 0.005 0.002 0.011 20

7: 0.003 0.002 0.002 0.003 0.955 0.002 0.003 0.004 0.003 0.003 0.003 0.001 0.003 0.003 0.004 0.003 0.002 0.003 47

8: 0.897 0.006 0.011 0.007 0.005 0.009 0.003 0.004 0.010 0.010 0.006 0.002 0.005 0.005 0.005 0.007 0.004 0.005 47

9: 0.007 0.004 0.008 0.005 0.003 0.916 0.005 0.005 0.005 0.004 0.004 0.003 0.006 0.006 0.005 0.004 0.004 0.006 45

10: 0.002 0.002 0.002 0.003 0.003 0.003 0.013 0.012 0.002 0.002 0.002 0.002 0.004 0.871 0.067 0.002 0.005 0.003 47

11: 0.003 0.018 0.006 0.004 0.013 0.006 0.006 0.004 0.004 0.018 0.080 0.002 0.005 0.003 0.004 0.017 0.004 0.801 22

12: 0.001 0.001 0.001 0.001 0.002 0.002 0.257 0.003 0.001 0.001 0.001 0.001 0.002 0.002 0.214 0.001 0.505 0.001 26

13: 0.001 0.001 0.001 0.001 0.001 0.002 0.060 0.001 0.001 0.002 0.001 0.915 0.002 0.002 0.002 0.001 0.002 0.001 21

14: 0.004 0.010 0.004 0.005 0.010 0.010 0.004 0.005 0.006 0.008 0.822 0.002 0.005 0.005 0.005 0.010 0.007 0.080 46

15: 0.002 0.010 0.002 0.007 0.041 0.002 0.002 0.003 0.003 0.005 0.071 0.002 0.003 0.003 0.004 0.038 0.003 0.800 23

16: 0.004 0.014 0.003 0.003 0.012 0.003 0.003 0.004 0.002 0.008 0.008 0.002 0.004 0.002 0.003 0.916 0.003 0.006 45

17: 0.003 0.002 0.003 0.961 0.002 0.002 0.001 0.002 0.006 0.003 0.002 0.001 0.002 0.002 0.002 0.002 0.002 0.002 23

18: 0.005 0.019 0.005 0.010 0.007 0.007 0.003 0.005 0.007 0.859 0.034 0.002 0.005 0.005 0.004 0.009 0.004 0.011 45
